# Correlating halide segregation of wide-bandgap perovskites with the methoxy group in organic hole-selective materials†

**DOI:** 10.1039/d4sc08810g

**Published:** 2025-04-01

**Authors:** Xiaoyu Ji, Yun Zhao, Xiaofeng Chen, Shuo Zhang, Liqing Zhan, Huidong Zhang, Weizhong Zheng, Wei-Hong Zhu, Yongzhen Wu

**Affiliations:** a Key Laboratory for Advanced Materials and Joint International Research Laboratory of Precision Chemistry and Molecular Engineering, Shanghai Key Laboratory of Functional Materials Chemistry, Feringa Nobel Prize Scientist Joint Research Center, Frontiers Science Center for Materiobiology and Dynamic Chemistry, Institute of Fine Chemicals, School of Chemistry and Molecular Engineering, East China University of Science and Technology Shanghai 200237 China wu.yongzhen@ecust.edu.cn shuozhang@ecust.edu.cn; b State Key Laboratory of Chemical Engineering, School of Chemical Engineering, East China University of Science and Technology Shanghai 200237 P. R. China; c Center of Photosensitive Chemicals Engineering, East China University of Science and Technology Shanghai P. R. China

## Abstract

Mixed-halide wide-bandgap (WBG) perovskites are widely used in constructing tandem photovoltaics, but their practical application is challenged by a phenomenon known as photo-induced halide segregation (PIHS), which is detrimental to the stability of the devices. The origin of PIHS is not fully understood yet, restricting the further advancement of mixed-halide WBG perovskites. Here, we report the serendipitous discovery that the PIHS of WBG perovskites is highly related to the presence of the methoxy group (MeO) in organic hole-selective materials (HSMs). Based on a model compound with triphenylamine as the hole-selecting group and cyanovinyl phosphonic acid as the anchoring group, we developed a series of HSMs which differed only in the substituent groups (MeO, methyl or hydrogen) on the triphenylamine. *In situ* photoluminescence (PL) measurements revealed that all HSMs with MeO groups exhibited severe PIHS, and this observation was further validated by commercial PACz-series HSMs. Temperature-dependent PL experiments and density functional theory calculations suggest that contact between the MeO group and perovskites reduces the diffusion energy barrier of the halide ion, thus accelerating the PIHS. Removing the MeO group from the HSMs not only improves the power conversion efficiency of 1.76 eV WBG perovskite solar cells from 19% to 21% but also enhances their operational stability, with T90 increasing from 180 h to 650 h. This work discloses PIHS caused by the molecular structure of HSMs and suggests that the MeO group should be avoided when designing interfacial materials for WBG-perovskite-related optoelectronic devices.

## Abstract 2

The methoxy group in hole-selective molecules can accelerate the photo-induced halide segregation in wide-bandgap perovskite, which due to the electrostatic interaction between methoxy group in hole-selective molecule and halide ions in perovskite.

## Graphical abstract

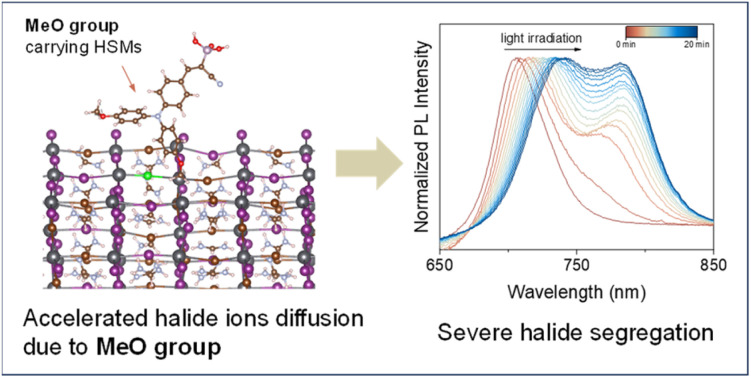

## Introduction

Wide-bandgap perovskite solar cells (WBG-PSCs) are promising cells for constructing efficient tandem photovoltaics to achieve high power conversion efficiency (PCE) at low cost.^1–3^ The most common approach to widening the bandgap is the incorporation of bromide (Br) instead of partial or all-iodide (I) in perovskites, which not only changes the valence band (VB) and conduction band (CB) energy levels but also decreases the materials' photostability due to the notorious photo-induced halide segregation (PIHS) observed in mixed-halide perovskites (especially when the ratio of Br exceeds 20%, which is common in tandems).^4–6^ Hole-selective materials (HSMs) in p-i-n structured WBG-PSCs play a crucial role in determining both the device performance and stability, as they not only affect the crystallization quality of the polycrystalline perovskite thin films but also impact the interfacial defect passivation and hole-extraction efficiency in devices.^1,3,7,8^ For instance, Steve Albrecht and coworkers found that a combination of fast hole extraction and minimized nonradiative recombination at the hole-selective interface, realized by carbazole-based self-assembled monolayer (SAM)-type HSMs, stabilized the WBG perovskite well with a bandgap of 1.68 eV under illumination.^1^

In our previous work, we developed cyanovinyl phosphonic acid (CPA)-based amphiphilic molecular HSMs with modulated highest occupied molecular orbital (HOMO) energy levels for 1.68 eV WBG-PSCs, which are frequently used in perovskite-silicon tandem solar cells.^8,9^ We found that the energy level alignment between the wide-bandgap perovskite and the hole-selective layer is very important to the photovoltaic performance. The application of double-methyl-substituted MePA-CPA to 1.68 eV perovskite achieved an unprecedentedly high open-circuit voltage (*V*_OC_) of 1.29 V and PCE of 22.3% under standard AM 1.5 sunlight, achieving the lowest *V*_OC_-deficit (<0.40 V) among reported WBG-PSCs. We also found that the assembly and redistribution of CPA-based amphiphilic molecules at the perovskite-substrate buried interface promoted the growth of a low-defect crystalline perovskite thin film and suppressed the PIHS to some extent.

In this work, we focus on WBG-PSCs with a bandgap of 1.76 eV, which are suitable for constructing all-perovskite and perovskite-organic tandem photovoltaics. A series of CPA-based molecular HSMs with meticulously tailored triphenylamine terminal groups were used to optimize the energy level alignment and reduce the *V*_OC_ deficit. The single-methyl-substituted Me/HPA-CPA shows an optimal energy level alignment with the 1.76 eV perovskite and results in the highest *V*_OC_ of 1.32 V among this group of structurally similar HSMs, confirming the importance of energy level alignment in WBG-PSCs. When the photo-stability of WBG perovskite films deposited on different HSMs was examined, we found an intriguing correlation between the PIHS and the presence of the methoxy group (MeO) in organic HSMs. We confirmed this serendipitous discovery by comparing the PIHS of WBG perovskite thin films deposited on a series of commercial and lab-produced carbazole-based HSMs. Temperature-dependent photoluminescence (PL) experiments and density functional theory calculations suggest that contact between the MeO group and perovskites reduces the diffusion energy barrier of halide ions, thus accelerating the PIHS. This work discloses that the molecular structure of HSMs caused PIHS and suggests that the MeO group should be avoided when designing interfacial materials for WBG-perovskites related optoelectronic devices.

## Results and discussion

### Energy level alignment improves performance of WBG-PSCs

**Fig. 1:**
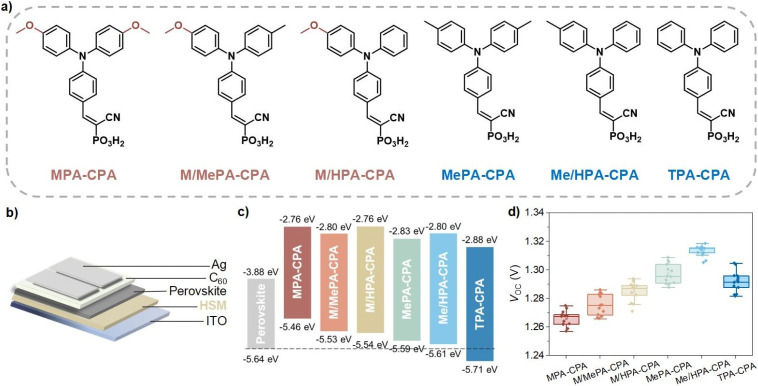
(a) Molecular structures of CPA-based HSMs, namely, (2-(4-(bis(4-methoxyphenyl)amino)phenyl)-1-cyanovinyl)phosphonic acid (MPA-CPA), (1-cyano-2-(4-((4-methoxyphenyl)(*p*-tolyl)amino)phenyl)vinyl)phosphonic acid (M/MePA-CPA), (1-cyano-2-(4-((4-methoxyphenyl)(phenyl)amino)phenyl)vinyl)phosphonic acid (M/HPA-CPA), (2-(4-(bis(4-methyl)amino)phenyl)-1-cyanovinyl)phosphonic acid (MePA-CPA), (1-cyano-2-(4-(phenyl(*p*-tolyl)amino)phenyl)vinyl)phosphonic acid (Me/HPA-CPA), and (cyano-2-(4-(di-*p*-tolylamino)phenyl)vinyl)phosphonic acid (TPA-CPA). (b) Schematic description of WBG-PSCs structure. (c) Energy diagram of perovskite and CPA-based HSMs. (d) Statistics of *V*_OC_ values obtained from *J*–*V* characteristics in reverse sweep modes for WBG-PSCs based on different CPA-based HSMs.

Compared to 1.68 eV perovskite, the ratio of Br in 1.76 eV perovskite is increased, which results in a deeper VB edge and more serious photo-stability issues.^4,10–12^ Traditional HSMs that were developed for normal bandgap (1.53–1.63 eV) or ∼1.68 eV perovskites are no longer competent for achieving highly efficient hole-extraction and defect passivation.^1,7,8,13^ Therefore, we planned to improve the energy level alignment at the hole-extraction interface for 1.76 eV WBG-PSCs by applying a series of CPA-based molecular HSMs with meticulously tailored triphenylamine terminal groups (Fig. 1a). Among these molecules, the symmetrically substituted MPA-CPA, MePA-CPA and TPA-CPA are known compounds that have been reported previously in the literature.^8,9^ The asymmetrically substituted M/MePA-CPA, M/HPA-CPA and Me/HPA-CPA are new compounds and their synthesis will be reported elsewhere. Their HOMO energy levels were measured using cyclic voltammetry scans (Fig. S1†) and are listed in Table S1.† As the substituent groups on the triphenylamine were changed from methoxy (MeO) to methyl (Me) and hydrogen (H), the electron-donating capability of the hole-selecting triphenylamine gradually decreased, resulting in a gradually deepened HOMO energy levels, as shown in Fig. 1c. Among these six HSMs, the HOMO energy level of Me/HPA-CAP (−5.61 eV) matches the VB edge (−5.64 eV) of the 1.76 eV perovskite better than the others, showing great promise for achieving higher photovoltaic performance (Fig. S2†).

The device architecture of the WBG-PSCs studied in this work is shown in Fig. 1b, and the detailed device fabrication methods and conditions can be found in the ESI.† Fig. S3a† shows the typical current density–voltage (*J*–*V*) curves of devices based on CPA-based HSMs, and the relevant photovoltaic parameters are listed in Table S2.† The short circuit current density (*J*_SC_) of these devices was almost identical, with values around 19.0 mA cm^−2^, which is coincident well with the external quantum efficiency (EQE) curves of the devices (Fig. S3b†). The statistical *V*_OC_ values of the WBG-PSCs based on different HSMs are shown in Fig. 1d. As expected, the Me/HPA-CPA based devices exhibited higher average *V*_OC_ (1.315 V) values than MPA-CPA (1.267 V), M/MePA-CPA (1.275 V), M/HPA-CPA (1.287 V), MePA-CPA (1.296 V) and TPA-CPA (1.292 V). Accordingly, WBG-PSCs based on Me/HPA-CPA achieved the highest power conversion efficiency (PCE) of 21% (Fig. S4†).

The changes in the ultraviolet-visible (UV-vis) absorption spectra of the perovskite films deposited on different HSMs were negligible (Fig. S5†). Additionally, we employed grazing-incidence wide-angle X-ray scattering (GIWAXS) and top-view scanning electron microscopy (SEM) to characterize the perovskite films deposited on these HSMs. The crystallization of perovskite films remained consistent across the CPA-based HSMs (Fig. S6†). All the perovskite films showed very similar morphology and grain size without apparent pinholes or cracks in top-view SEM images (Fig. S7†). We further measured the photoluminescence quantum yield (PLQY) of these samples (Fig. S8†). The quasi-Fermi level splitting (QFLS) was calculated from the PLQY to quantify the *V*_OC_ potential for the WBG-PSCs, with the corresponding data listed in Table S3.† It is apparent that the 1.76 eV perovskite films deposited on Me/HPA-CPA exhibited the highest QFLS values (1.36 eV) among the CPA-based HSMs, which is in good agreement with their highest *V*_OC_ in complete devices. Moreover, the perovskite films deposited on Me/HPA-CPA also showed longer Shockley–Read–Hall (SRH) lifetimes than the others. These results suggest that energy level alignment is a universally useful strategy to improve the performance of WBG-PSCs by reducing nonradiative recombination losses at the hole-collection interface.

### Discovery of MeO group accelerated PIHS

**Fig. 2:**
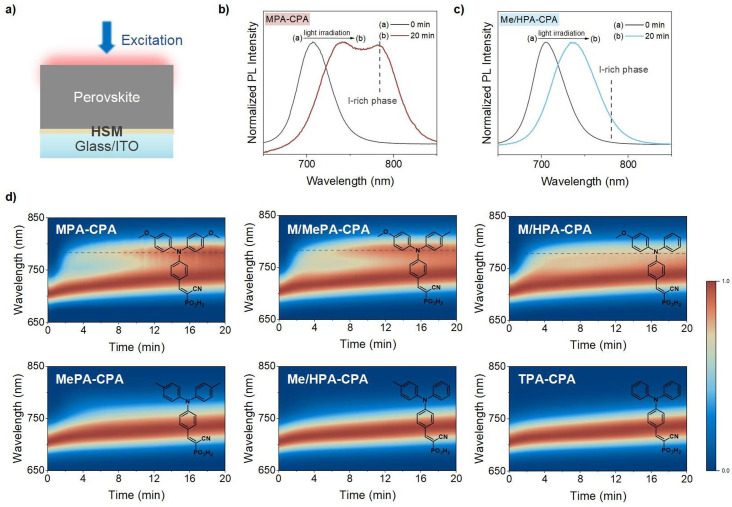
(a) Schematic description of photoluminescence (PL) experiment. PL spectra of 1.76 eV perovskite deposited on (b) MPA-CPA and (c) Me/HPA-CPA after light irradiation for 20 min. (d) Evolution of PL spectra of 1.76 eV perovskite tracked over 20 min utilizing MPA-CPA, M/MePA-CPA, M/HPA-CPA, MePA-CPA, Me/HPA-CPA and TPA-CPA as HSMs. The scale bar represents the normalized PL intensity of the perovskite films, and the dotted line represents the emergence of I-rich phases.

The high molar ratio of Br (∼30%) in 1.76 eV perovskite typically results in prominent PIHS under light irradiation.^10–12,14–17^ As an increase in PL intensity at lower photon energies indicates the formation of iodide-rich (I-rich) phases, photoluminescence (PL) measurement have commonly been used to characterize the extent to which PIHS has occurred in WBG perovskites.^18,19^ We employed an *in situ* PL measurement to explore the dynamic generation and evolution of the PIHS in the 1.76 eV perovskite films deposited on different HSMs. Samples were fabricated with an indium tin oxide (ITO)/HSM/perovskite structure, as shown in Fig. 2a.

Before light irradiation, all the pristine perovskite films exhibited PL emission with a peak at ∼704 nm (Fig. S9†). After continuous light irradiation of these samples for 20 min, we observed in general two types of variation in the PL spectra. One type was a red-shift in the PL emission plus the generation of a new emission with a peak at ∼780 nm, as shown in Fig. 2b, while the other type was a simple red-shift in the PL emission without the generation of new peak at 780 nm (Fig. 2c). The recovery of the PL under dark conditions confirmed the occurrence of reversible PIHS rather than degradation in the perovskite films (Fig. S10†).

To determine the degree of PIHS in these two types, the PL spectra of the perovskites under light irradiation were measured for 90 min, and the growth rate constants of the perovskite films were calculated. The induced emission at 775 nm was tracked to determine the rate of PIHS (Fig. S11†). All kinetic traces were fitted with monoexponential functions to calculate the rate constants. The rate constant of PIHS decreased from *k* ≈ 8.2 × 10^−3^ s^−1^ for the former type to *k* ≈ 1.1 × 10^−3^ s^−1^ for the latter type. Apparently, the degree of PIHS is more severe for the former type (Fig. S12†).

To visualize the PIHS in these two types of samples, we performed cross-sectional SEM energy-dispersive X-ray spectroscopy (SEM-EDS) to acquire elemental mapping of the WBG perovskite films (MPA-CPA and Me/HPA-CPA as HSMs) after light irradiation for 30 min. As shown in Fig. S13,† an accumulation of iodide and no obvious accumulation of bromide were observed on the surface of the MPA-CPA based perovskite film. However, a uniform distribution of bromide and iodide was observed for the Me/HPA-CPA based perovskite film. These observations are consistent with the PL evolution, indicating the generation of a new emission at ∼780 nm (I-rich phase) in perovskites based on MPA-CPA.

The dynamic evolution of the PL emission is presented in Fig. 2d, in which we found an interesting tendency, namely, that all HSMs with MeO groups (MPA-CPA, M/MePA-CPA and M/HPA-CPA) aggravated the PIHS and led to the generation of a new emission at ∼780 nm. Conversely, the HSMs without MeO groups only experienced a red-shift in PL emission. It seems that the presence of MeO groups in organic HSMs accelerates the PIHS.

**Fig. 3:**
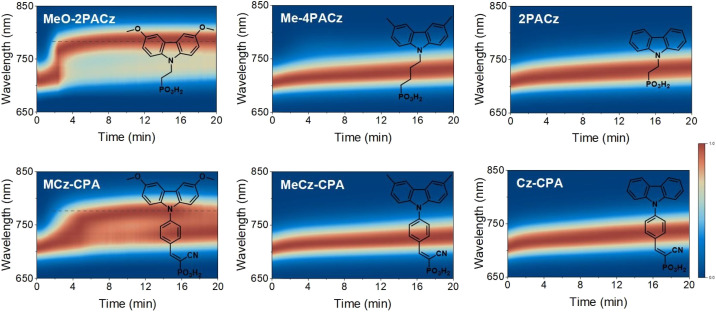
Time-dependent PL spectra of 1.76 eV perovskite tracked over 20 min utilizing MeO-2PACz, Me-4PACz, 2PACz, MCz-CPA, MeCz-CPA and Cz-CPA as HSMs. The scale bar represents the normalized PL intensity of perovskite films deposited on different HSMs, and the dotted line represents the emergence of I-rich phases.

To verify this hypothesis, we further compared the PIHS of the perovskite films on a series of carbazole-based HSMs (Fig. 3), including the commercially available alkyl-linked PACz series (MeO-2PACz, Me-4PACz and 2PACz) and our laboratory-produced phenyl-linked CPA series (MCz-CPA, MeCz-CPA and Cz-CPA). The PL growth rates of perovskite films based on carbazole-based HSMs were also calculated to evaluate the degree of PIHS (Fig. S14†). We observed a very similar trend, both HSMs with MeO groups (MeO-2PACz and MCz-CPA) being associated with more severe PIHS (Fig. S15†). Notably, the HOMO energy levels of these molecular HSMs were distributed broadly from −5.2 eV to −5.8 eV, suggesting a large variation of hole-extraction efficiency due to the significantly varied energy level alignment. However, the degree of PIHS was not always consistent with the energy level alignment. For example, the TPA-CPA and Cz-CPA, which had the deepest HOMO levels (obviously unmatched with the VB of the perovskite), did not show serious PIHS. Additionally, we modified the HOMO energy level of MePA-CPA by chemically reducing the vinyl bond, and found that it did not significantly change the degree of PIHS (Fig. S16†).

Further, the presence of traps in the perovskite films can also be relevant for PIHS. We measured the light intensity (*P*)-dependent *V*_OC_ to investigate the effect of the underlayer on traps in the perovskite (Fig. S17†). The consistent slopes of the *V*_OC_–*P* curves indicate a similar level of traps in these perovskite films, which indicates that the different PIHS does not arise from a different degree of traps caused by the HSM underlayer. Additionally, we explored the potential impact of cyano and phosphonic acid groups in the HSMs on PIHS to isolate the effect of the MeO group (Fig. S18†). The evolution of the PL spectra indicates that the cyano and phosphonic acid groups had little effect on the PIHS of the perovskite, which further confirmed the specific effect of the MeO group.

These results provided an important hint regarding the factors that facilitate or suppress PIHS in WBG perovskites. Previous studies have revealed that fast hole extraction is a key factor to weaken the PIHS,^1,11,19–22^ while our results suggest that certain chemical structures (such as MeO) and/or the microenvironment at the interface between the perovskite and charge-selective materials can be additional factors that greatly impact the PIHS of WBG perovskites.

### Mechanical studies of MeO-group-accelerated PIHS

We recorded the *in situ* PL of perovskites under continuous light irradiation at different temperatures (15, 25, 30, 40, 50, 60 and 65 °C) and examined the PL intensity change at 775 nm (Fig. S19†). The curves were monoexponentially fitted to calculate the PL growth rates,^23,24^ which are listed in Table S4.† It is evident that the rate of PIHS increases significantly as the temperature rises.

**Fig. 4:**
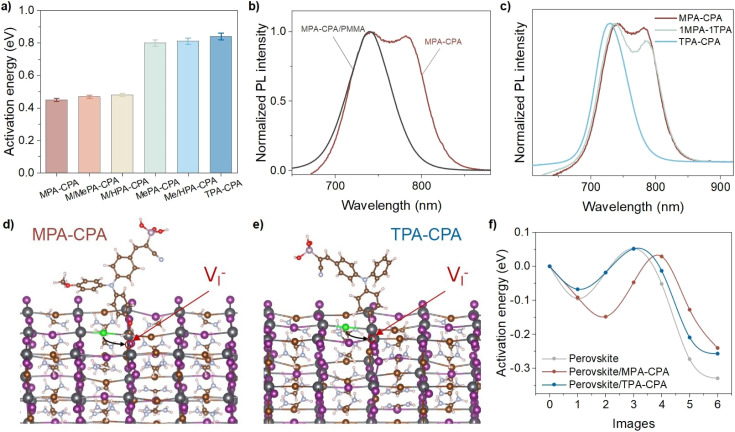
(a) Activation energy of PIHS in perovskite films deposited on different HSMs. (b) Normalized PL intensity of perovskite deposited on MPA-CPA or MPA-CPA/PMMA after light irradiation for 20 min. (c) PL spectra of 1.76 eV perovskite films deposited on mixed HSMs of MPA-CPA and TPA-CPA after light irradiation for 20 min. The initial interfacial structures for the (d) MPA-CPA/perovskite model and (e) TPA-CPA/perovskite model containing halide vacancies (V_I_^−^). The MeO groups of the molecule are at a height of 1.554 Å above the top of the surface. (f) DFT calculation of the ion diffusion activation energy for the migration of halide ions to adjacent halide vacancies.

This temperature dependence was further analyzed using the Arrhenius equation:1
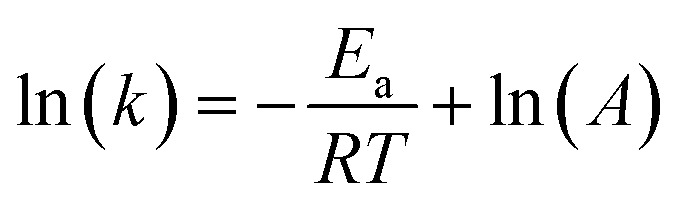
where *A* is a preexponential constant and *R* is the gas constant (*R* = 8.314 J mol^−1^ K^−1^). The plot of ln(*k*) *versus* inverse temperature in Fig. S20† exhibits a linear relationship. The activation energies of PIHS are determined from the slope of the plots, being approximately 0.47 and 0.81 eV for perovskites deposited on MeO-containing and MeO-free HSMs, respectively (Fig. 4a). These values are similar to the activation energies reported in the literature.^23–26^ The lower thermodynamic activation barrier might be related to the contact between MeO and the perovskite.

To evaluate the impact of direct contact between the MeO group and perovskite on the PIHS, we deposited a thin layer of poly(methyl methacrylate) (PMMA) on top of the HSMs before the deposition of the WBG perovskite and examined the resulting photostability (Fig. S21†). As shown in Fig. 4b, the insertion of PMMA obviously suppressed the formation of the new emission at around 780 nm in the PL spectra after light irradiation for 20 min. We further used a thin layer of tin oxide (SnO_2_) deposited *via* the atomic layer deposition technique instead of PMMA in the above test and found that the PIHS was again greatly suppressed (Fig. S22†). The PL intensity ratios of the I-rich phase to the mixed-halide phase among the different perovskite films indicated the suppressed PIHS of the HSM/PMMA/perovskite and HSM/SnO_2_/perovskite samples compared to the HSM/perovskite sample.

To test the sensitivity of PIHS to the presence of the MeO group in HSMs, we used mixtures of MPA-CPA and TPA-CPA to deposit the hole-selective layer and examine the photo-stability of the WBG perovskites on top of them. As shown in Fig. 4c and S23,† the double-peak emission appeared for perovskites deposited on HSMs containing MeO substituents, indicating that the existence of MeO substituents accelerates the halide ion migration and separation.

Density functional theory (DFT) calculations were employed to understand the role of the MeO group in promoting halide ion migration. The diffusion paths of halide ions were calculated at the surface of perovskites containing halide vacancies (V_I_^−^) in contact with MPA-CPA and TPA-CPA (Fig. 4d, e and S24–S26†). The ion diffusion activation energies of halide ions diffusing in the perovskite are shown in Fig. 4f. In the presence of MPA-CPA, the ion diffusion activation energy (0.03 eV) of the halide ions in perovskite is lower than that in the TPA-CPA/perovskite model (0.05 eV) and perovskite model (0.05 eV), confirming that the presence of MeO groups reduces the energy barrier for halide ion diffusion and promotes the migration of halide ions. Based on analysis of the ion diffusion path, the repulsion of the electron-rich MeO group makes it easier for nearby halide ions to deviate from their original positions (Fig. S27†), corresponding to images 0–2 in Fig. S25.† Simultaneously, the diffusion activation energy of the TPA-CPA/perovskite and pure perovskite models did not change much at any position, further proving the specific effect of the MeO group on PIHS. In addition, the diffusion activation energy (−0.24 eV) of halide ions at V_I_^−^ is slightly higher than that of TPA-CPA/perovskite model (−0.26 eV) and perovskite model (−0.33 eV). The higher diffusion activation energy indicates that halide ions in this state are more inclined to deviate from this position and continue to diffuse, which may result from the greater repulsive effect of the electron-rich MeO groups on halide ions. We concluded that the existence of MeO groups affects the activation energy of PIHS, accelerating the halide ion diffusion and migration in perovskite films based on MeO-containing HSMs.

### Impact of PIHS on WBG-PSC stability

**Fig. 5:**
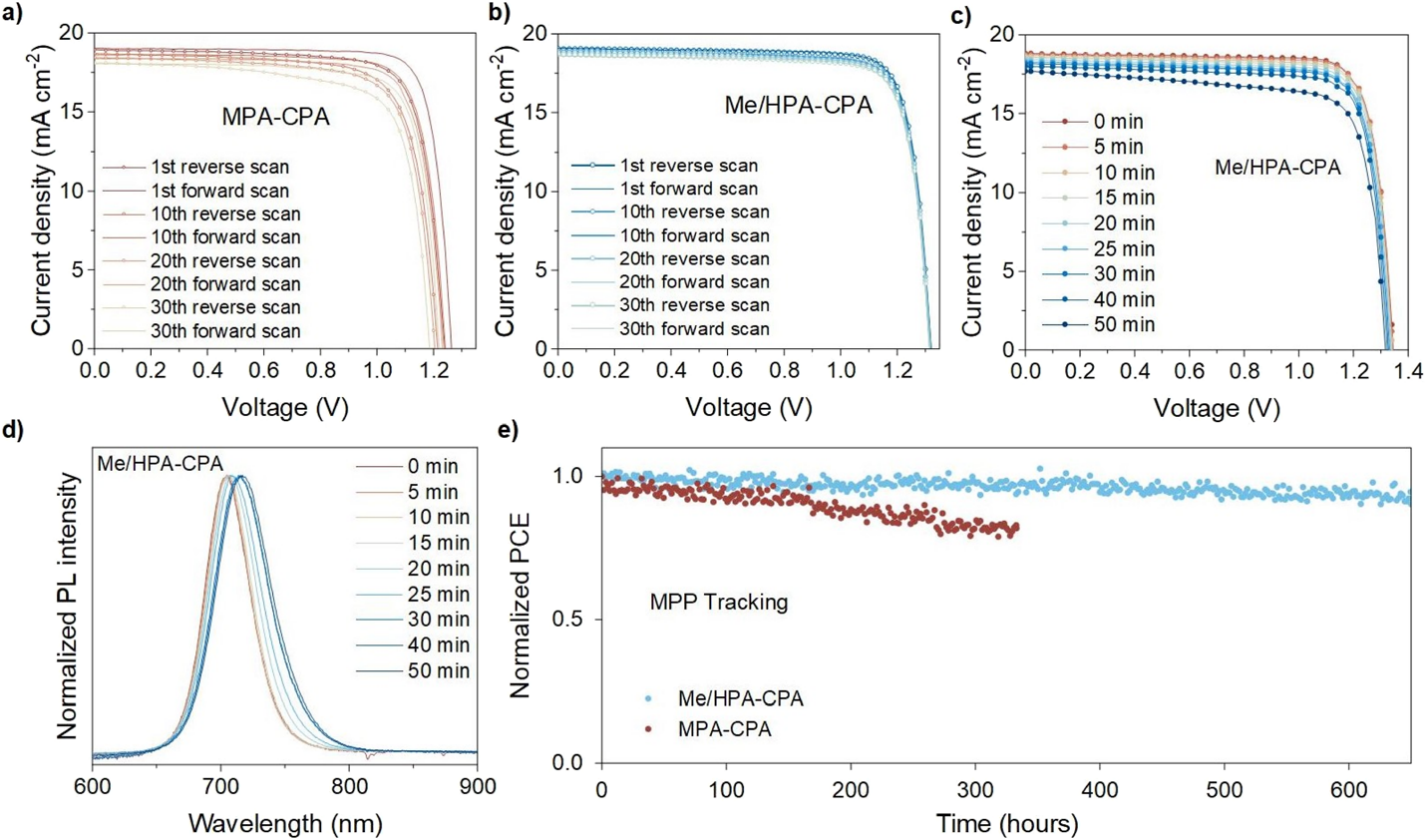
*J*–*V* curves of 30 forward and reverse scan cycles with a scan rate of 0.10 V s^−1^ for the devices based on (a) MPA-CPA and (b) Me/HPA-CPA. Evolution of the (c) *J*–*V* curves and (d) PL spectra of Me/HPA-CPA based devices tracked over 50 min under light irradiation (xenon lamp, 1 sun). (e) Operational stability of WBG-PSCs based on MPA-CPA and Me/HPA-CPA under nitrogen conditions. The initial PCEs of the MPA-CPA and Me/HPA-CPA based WBG-PSCs were 18.9% and 20.3%, respectively.

To examine the impact of the different degrees of PIHS on the stability of the complete devices, repeated *J*–*V* scans in both the forward and reverse directions were carried out to investigate the variation of the hysteric behavior of WBG-PSCs based on MPA-CPA and Me/HPA-CPA, as the current–voltage hysteresis is related to ionic migration in perovskites.^27–29^ Hysteresis was observed in the first scan cycle for the devices based on MPA-CPA, and the hysteretic behavior persisted as the number of scan cycles increased (Fig. 5a). In contrast, no observable hysteresis was recorded over 30 scan cycles for the WBG-PSCs based on Me/HPA-CPA (Fig. 5b). The cross-section elemental mapping of Me/HPA-CPA based WBG-PSCs after repeated *J*–*V* scans showed a uniform distribution of bromide and iodide in the perovskite layer (Fig. S28†). The PL emission of these WBG-PSCs in Fig. S28† is also almost the same as initially, indicating improved halide-composition stability. These results suggest that the lack of observable hysteresis in the Me/HPA-CPA based devices may be related to the suppressed PIHS in the perovskite films. Fig. S29† shows that devices based on Me/HPA-CPA have improved stability compared to devices based on MPA-CPA, which had PCEs maintaining 97% of their initial efficiency after 30 *J*–*V* scans.

We measured the device performance and PL spectra of Me/HPA-CPA and MPA-CPA based WBG-PSCs under light irradiation (Fig. 5c, d and S30†). A slight red-shift in the PL emission appeared in the Me/HPA-CPA based devices under illumination, while a red-shift in PL emission plus the generation of ∼780 nm emission appeared for the MPA-CPA based devices; these results were similar to the PL evolution of the perovskite films. The *V*_OC_ values of the devices gradually decreased with increasing illumination time, which may be related to the PIHS in the perovskite. Fig. S31† summarizes the variation in the *V*_OC_ and band-gap (*E*_g_) of the Me/HPA-CPA based devices with irradiation time. During the first 15 min of illumination, the decrease in *V*_OC_ is basically consistent with the reduction in *E*_g_. After 15 min of illumination, the decrease of *V*_OC_ is slower than that of *E*_g_, indicating that the decrease in *V*_OC_ is not dominated by PIHS in this process. Hence, we measured the time-dependent PLQY for ITO/Me/HPA-CPA/1.76 eV perovskite/passivation/C_60_ stacks under light irradiation to quantify the *V*_OC_ potential of the devices (Fig. S32†). The QFLS of the samples at different irradiation times was evaluated and calculated. The results show that the QFLS values decreased gradually over the first 12 min, which is consistent with the decrease observed for the Me/HPA-CPA based devices during the first 15 min. In addition, the QFLS values increased slightly during the last 40 min, indicating reduced nonradiative recombination losses in the perovskites with smaller *E*_g_ values after PIHS, so the reduction of *V*_OC_ is slower than that of *E*_g_. As a result, we concluded that PIHS may lead to a rapid degradation of device performance under short-term illumination (less than 15 min), and the damping of long-term illumination stability of devices would be slowed down by the reduced nonradiative recombination losses in the perovskites after PIHS.

Subsequently, the operational stability of WBG-PSCs based on MPA-CPA and Me/HPA-CPA was studied by employing the International Summit on Organic Photovoltaic Stability (ISOS) standards.^30^ As shown in Fig. 5e and S33,† the Me/HPA-CPA based devices maintained 90% of their initial PCE values after ∼650 h. In contrast, the MPA-CPA based WBG-PSCs only retained 80% of their initial performance after ∼340 h under the same testing conditions. The greater operational stability of the WBG-PSCs based on Me/HPA-CPA was consistent with the suppression of PIHS in the perovskites, demonstrating that HSMs without MeO substituents remarkably increase the stability of mixed-halide WBG perovskite devices.

## Conclusions

In this work, we found that the presence of MeO groups in HSMs promotes the PIHS of WBG perovskites under light irradiation, and thoroughly studied the reasons underlying the observed phenomenon. Based on analysis of WBG perovskite films deposited on different HSMs, we confirmed that the presence of MeO groups in the hole-selective molecular structure is an important factor in the severe PIHS in perovskites deposited on HSMs with MeO groups. The presence of MeO groups reduces the energy barrier of halide ion diffusion and makes halide ions deviate more easily from their position according to the calculation results. The removal of MeO groups from the molecular structure of HSMs can fundamentally mitigate the accelerated PIHS in WBG perovskite films and improve the operational stability of WBG-PSCs. We propose that the molecular structure of the HSMs plays a key role in the photostability of the perovskite, and most importantly, in WBG perovskites applied in perovskite-based tandem photovoltaic cells.

## Data availability

The data supporting this article have been included as part of the ESI.†

## Author contributions

X. J. conceived the project, which was supervised by Y. W. and W. Zhu, Y. Z. and S. Z. synthesized CPA-based molecules, X. C. carried out DFT calculation, which was supervised by W. Zheng, L. Z. synthesized Re-MePA-CPA molecules, X. J. conducted PSC device characterizations and wrote the manuscript with the assistance of Y. Z., X. C., S. Z. and H. Z., and all authors participated in data analysis and discussion.

## Conflicts of interest

There are no conflicts to declare.

## Supplementary Material

SC-016-D4SC08810G-s001
