# Supplementary material for: Correlating halide segregation of wide-bandgap perovskites with the methoxy group in organic hole-selective materials
Source: Chem Sci. 2025 Apr 1;16(19):8569–76. doi: 10.1039/d4sc08810g (PMC11998020; doi:10.1039/d4sc08810g)
Supplement: SC-016-D4SC08810G-s001 [file SC-016-D4SC08810G-s001.pdf]

Supplementary Information (SI) for Chemical Science.
This journal is © The Royal Society of Chemistry 2025

Supplementary Information

Correlating halide segregation of wide-bandgap perovskites with methoxy group in organic hole-selective materials

Xiaoyu Ji, Yun Zhao, Xiaofeng Chen, Shuo Zhang, Liqing Zhan, Huidong Zhang, Weizhong Zheng, Wei-Hong Zhu and Yongzhen Wu

Materials

Patterned indium tin oxide (ITO) (~10 Ω/sq) coated glass was purchased from Beijing HuaMin New Materials Technology Co., Ltd. Formamidine hydroiodide (FAI), and lead iodide (PbI_2) were sourced from Advanced Election Technology Co., Ltd. Lead bromide ($PbBr_2$) was obtained from Energy Chemical. Cesium iodide (CsI), lead chloride ($PbCl_2$), phenethylphosphonic acid (Ph-PA) and 3-phenylpropanenitrile (Ph-CN) were acquired from Shanghai Macklin Biochemical Technology Co., Ltd. Methylamine hydrobromide (MABr) and 2-phenylethylamine hydroiodide (PEAI) were purchased from Xi'an Yuri Solar Co., Ltd. Methylammonium chloride (MACI) was purchased from Alfa Aesar. C_{60} was sourced from Nanjing/Jiangsu XFNANO Materials Tech Co., Ltd. Bathocuproine (BCP), [2-(3,6-dimethoxy-9*H*-carbazol-9-yl)ethyl]phosphonic acid (MeO-2PACz), [2-(9*H*-carbazol-9-yl)ethyl]phosphonic acid (2PACz) and [4-(3,6-dimethyl-9*H*-carbazol-9-yl)butyl]phosphonic acid (Me-4PACz) were obtained from Tokyo Chemical Industry (TCI). 4-(Diphenylamino)benzonitrile (TPA-CN) was sourced from leyan, shanghai, China. All the anhydrous solvents were procured from Acros Organics. All materials were utilized without further treatment. The hole-selective materials (HSMs) of 2-(4-(bis(4-methoxyphenyl)amino)phenyl)-1-cyanovinyl)phosphonic acid (**MPA-CPA**), (2-(4-(bis(4-methyl)amino)phenyl)-1-cyanovinyl)phosphonic acid (**MePA-CPA**), cyano-2-(4-(di-ptolylamino)phenyl)vinyl)phosphonic acid (**TPA-CPA**), (1-cyano-2-(4-(3,6-dimethoxy-9H-carbazol-9-yl)phenyl)vinyl)phosphonic acid (**MCz-CPA**), (1-cyano-2-(4-(3,6-dimethyl-9H-carbazol-9-yl)phenyl)vinyl)phosphonic acid (**MeCz-CPA**) and (2-(4-(9H-carbazol-9-yl)phenyl)-1-cyanovinyl)phosphonic acid (**Cz-CPA**) were synthesized as reported in our previous studies. The HSMs of (1-cyano-2-(4-((4-methoxyphenyl)(p-tolyl)amino)phenyl)vinyl)phosphonic acid (**M/MePA-CPA**), (1-cyano-2-(4-((4-methoxyphenyl)(phenyl)amino)phenyl)vinyl)phosphonic acid (**M/HPA-CPA**) and (1-cyano-2-(4-(phenyl(p-tolyl)amino)phenyl)vinyl)phosphonic acid (**Me/HPA-CPA**) and (1-cyano-2-(4-(di-p-tolylamino)phenyl)ethyl)phosphonic acid (**Re-MePA-CPA**), and the small molecule of (1-cyano-2-phenylvinyl)phosphonic acid (Ph-CPA) were synthesized and would be reported in future works.

WBG perovskite solar cell fabrication

The ITO glasses were washed with soapy water, deionized water, ethanol and acetone in sequence. Subsequently, the cleaned substrates were subjected to a 20-minute treatment with ultraviolet (UV) ozone and immediately transferred into a nitrogen-filled glovebox. For hole-selective layer (HSL) deposition, solutions of all hole-selective materials (HSMs) at a concentration of 1.0 mg mL^{-1} in anhydrous ethanol were used. The HSMs solutions were dropped onto ITO substrates at 3000 rpm for 30 seconds. After spin-coating, the substrates annealed at 100 °C for 10 minutes. The perovskite precursor solution ($Cs_{0.05}FA_{0.80}MA_{0.15}Pb(I_{0.70}Br_{0.30})_3$+5%$MAPbCl_3$) was stirred at 60 °C for 12 hours. Subsequently, 75 μL of the perovskite precursor solution was spin-coated onto the HSL using a two-step spin-coating procedure. The first step involved spinning at 2000 rpm for 10 seconds, followed by the second step at 6000 rpm for 40 seconds. At the 25 seconds into the second step, 200 μL of chlorobenzene was slowly dripped onto the film. The resulting perovskite film was annealed at 100°C for 30 minutes. The PEAI solution was dissolved in isopropanol (IPA) with a concentration of 1 mg mL^{-1}, and spin-coated onto perovskite film at 6000 rpm for 30 seconds without subsequent annealing. Subsequently, 20 nm C_{60}, 7 nm BCP, and 100 nm silver (Ag) electrode were thermally evaporated, respectively.

Characterization

Cyclic voltammograms (CV) were recorded employing a CHI660E electrochemical workstation (Chenhua Co. Ltd, Shanghai, China), using a three-electrode cell comprising a glassy carbon working electrode, a platinum (Pt) wire auxiliary electrode, and a saturated calomel reference electrode (SCE) in a saturated KCl solution. Notably, 0.1 M tetrabutylammonium hexaflourophosphate ($TBAPF_6$) was used in dichloromethane as the supporting electrolyte. Ultraviolet photoelectron spectroscopy (UPS) measurements were performed within an ultrahigh vacuum surface analysis system equipped with the ThermoFisher Nexsa. UPS employed He I 21.22 eV as the excitation source. Ultraviolet-visible (UV-vis) absorption spectra of solutions and films were tracked by a UV-2600 spectrometer (Shimadzu). Scanning electron microscopy (SEM) images and corresponding energy-dispersive X-ray spectroscopy (SEM-EDS) elemental mappings were measured by using a field-emission scanning electron microscope (Helios G4 UC, ThermoFisher Scientific). Time-resolved photoluminescence (TRPL) decay lifetimes were measured with an Edinburgh Instruments Fluorescence Spectrometer (FLS1000, under 510 nm excitation for TRPL). Time-dependent photoluminescence spectra were collected using a home-built facility, where an excitation light (405 nm, 1-sun equivalent) was introduced to the sample, and the PL spectra were monitored using QE Pro (Ocean Optics) spectrometer. The photoluminescence quantum yield (PLQY) of perovskite films was measured under integrating sphere mode in the air (Ocean QY) with a 405 nm light-emitting diode (LED) light source used as excitation. Grazing-incidence wide-angle X-ray scattering (GIWAXS) measurements were conducted at the BL14B1 of Shanghai Synchrotron Radiation Facility (SSRF), Shanghai, China, using beam energy of 10 keV (λ = 1.24 Å) and a Mar 225 detector. GIWAXS-Tools was utilized for necessary corrections of GIWAXS raw patterns.

Current density-voltage (*J-V*) characteristics of WBG-PSCs were measured using a Keithley 2400 Source Meter under xenon lamp with simulated AM 1.5G solar illumination condition (Zolix Instrument Co., Ltd). The light intensity was calibrated to 100 mW/cm^2 by standard silicon cell prior to testing. The *J-V* scans for WBG-PSCs were conducted in both forward and reverse sweep modes, spanning voltage ranges from -0.1 V to 1.34 V and from 1.34 V to -0.1 V, respectively, with a scan step of 0.02 V (or 0.01 V) and a delay time of 50 milliseconds. The active area of the devices was defined using a metal aperture, resulting in an active area of 0.0805 cm^2 (while the device contact area remained at 0.13 cm^2). External quantum efficiency (EQE) measurements were conducted using SCS600 Solar Cell Quantum Efficiency Measurement System (Zolix Instrument Co., Ltd) with a xenon arc lamp. The system was calibrated using a silicon reference cell with a known spectral response before measurement. The operational stability of unencapsulated devices was conducted by multi-channel solar cell stability test system (Wuhan 91PVKSolar Technology Co. Ltd, China). Operational stability assessments were carried out by placing the devices within the nitrogen chamber subjected to continuous white LED illumination (equal to 1 sun intensity), under controlled humidity (30-45% relative humidity) condition. The maximum power point (MPP) tracking was executed based on reverse scans every 12 hours.

Calculation of the quasi-Fermi level splitting (QFLS)

We calculated the internal electron-hole quasi-Fermi level splitting (QFLS) from PLQY results, employing the following equation:

$$\mathrm{QFLS} = k_B T \times \ln(\mathrm{PLQY} \times J_G / J_0^{rad})$$

where J_G is the generated current density at 1 sun illumination, approximated as the short-circuit current density of the complete solar cell. J_0^{rad} is the radiative recombination current in the dark and is estimated by integrating the overlap of the EQE of the full device with the black body radiative spectrum at 300 K over the energy range:

$$J_0^{rad} = q\int_0^{\infty} EQE_{PV}(E)\emptyset_{BB}(E)dE$$

The black body radiative spectrum, $\emptyset_{BB}(E)$, is defined as:

$$\emptyset_{BB}(E) = \frac{2\pi E^2}{h^3c^2}\frac{1}{\exp\left(\frac{E}{k_BT}\right) - 1}$$

Where E is the photo energy, h is the Plank constant, and c is the light speed in Vacuum. The value of J_0^{rad} is 6.718 $\times 10^{-23}$ A m^{-2}, derived from the EQE spectrum of the full device and the blackbody radiative spectrum.

Density functional theory (DFT) calculation

Calculations were performed within the DFT framework as implemented in Vienna Ab Initio Simulation Package (VASP) with the projected augmented wave (PAW) pseudopotential.[1,2] The generalized gradient approximation with the Perdew–Burke–Ernzerhof (PBE) functional was employed to describe exchange and correlation effects[3]. DFT-D3 method was used for the van der Waals correction. The plane-wave cutoff energy was 520 eV. The energy and force convergence criteria were set to 10^{-6} and 0.05 eV $Å^{-1}$, respectively. The Brillouin zone was sampled with Γ-centred k-mesh densities of more than 2π × 0.02 $Å^{-1}$ for geometry optimization and energy-related calculations. A 3-layer 3 × 3 supercell (approximately 19 Å × 18 Å) of $FA_{0.81}MA_{0.19}PbI_2Br$ (010) was used as a simplified surface model for $Cs_{0.05}FA_{0.80}MA_{0.15}Pb(I_{0.70}Br_{0.30})_3$+5%$MAPbCl_3$. Models of iodide-defect (I-defect) vacancy (initial positions) as well as the situation where iodide (I) near the I vacancy migrates to the I vacancy (final positions) were constructed. A vacuum layer with a thickness of 20 Å was added to avoid artificial interactions between the nanosheet and its images. Subsequently, the ion migration path models were generated by utilizing the nebmake.pl script that comes with the VTST, and calculations were carried out using the Climbing Image Nudged Elastic Band (CI-NEB) method[4]. The final structure was illustrated with VESTA software.

The plane-averaged charge density difference (PCDD) Δρ(z) is defined as Equation:

$$\Delta\rho(z) = \rho(\text{HSM-slab})(z) - \rho(\text{HSM})(z) - \rho(\text{slab})(z)$$

where ρ(HSM-slab) is the plane-averaged charge density of the $FA_{0.81}MA_{0.19}PbI_2Br$ slab contacted with **MPA-CPA** or **TPA-CPA** along z axis, ρ(HSM) is the plane-averaged charge density of **MPA-CPA** or **TPA-CPA** along z axis, and ρ(slab) the plane-averaged charge density of the $FA_{0.81}MA_{0.19}PbI_2Br$ slab along z axis. The Δρ(z) is the plane-averaged charge density difference along z axis, with unit of e/Å.

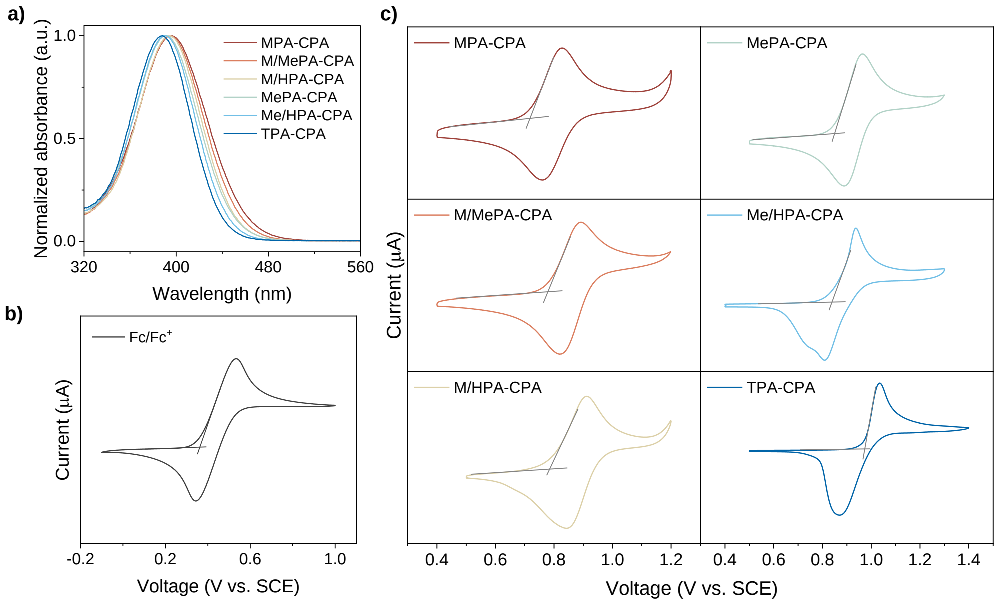

Figure S1. a) UV-vis absorption spectra of **MPA-CPA**, **M/MePA-CPA**, **M/HPA-CPA**, **MePA-CPA**, **Me/HPA-CPA** and **TPA-CPA** measured in ethanol. c) CV curves of CPA-based hole-selective materials (HSMs) measured in CH_2Cl_2, with ferrocene (Fc/Fc^+) serving as the external standard b).

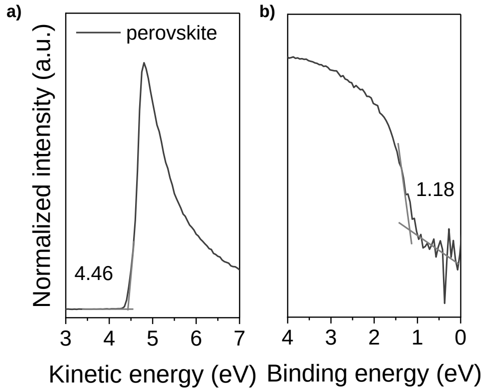

Figure S2. UPS of 1.76 eV perovskite film. a) The secondary electron cutoff region. b) Valence band onset. The valence-band maximum of perovskite was calculated to be -5.64 eV.

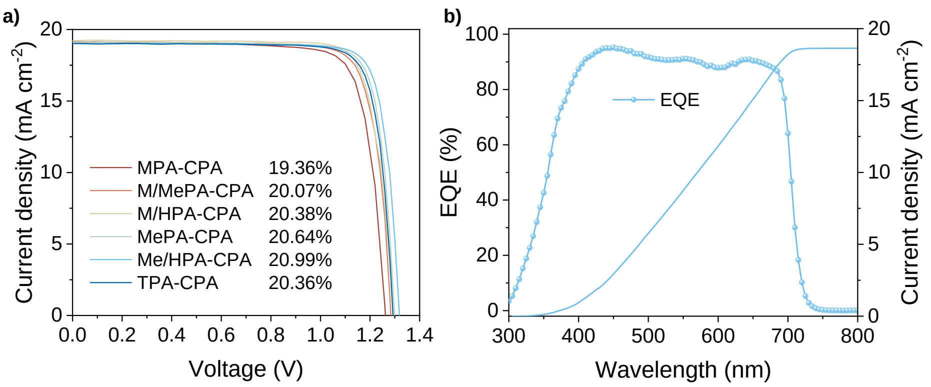

Figure S3. a) Typical *J-V* curves of the champion wide-bandgap perovskite solar cells (WBG-PSCs) based on CPA-based HSMs with an aperture area of 0.0805 cm^2. b) Typical EQE curve of WBG-PSC, with integrated J_{SC} values of 18.63 mA cm^{-2}.

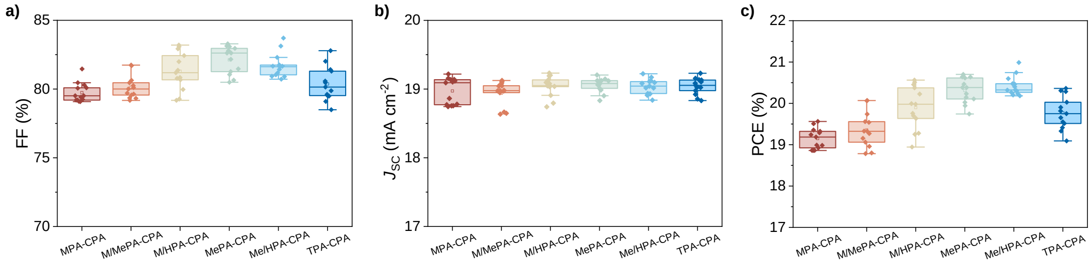

Figure S4. Statistics of fill factor (FF), short circuit current density (J_{SC}) and power conversion efficiency (PCE) values obtained from *J-V* characteristic in reverse sweep modes for WBG-PSCs based on different CPA-based HSMs.

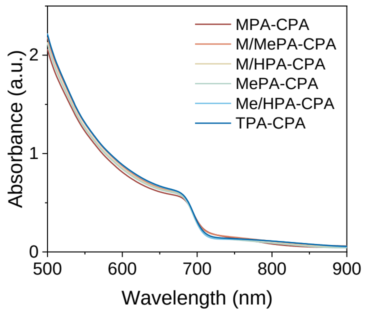

Figure S5. UV-vis absorption spectra of perovskite films deposited on CPA-based HSMs.

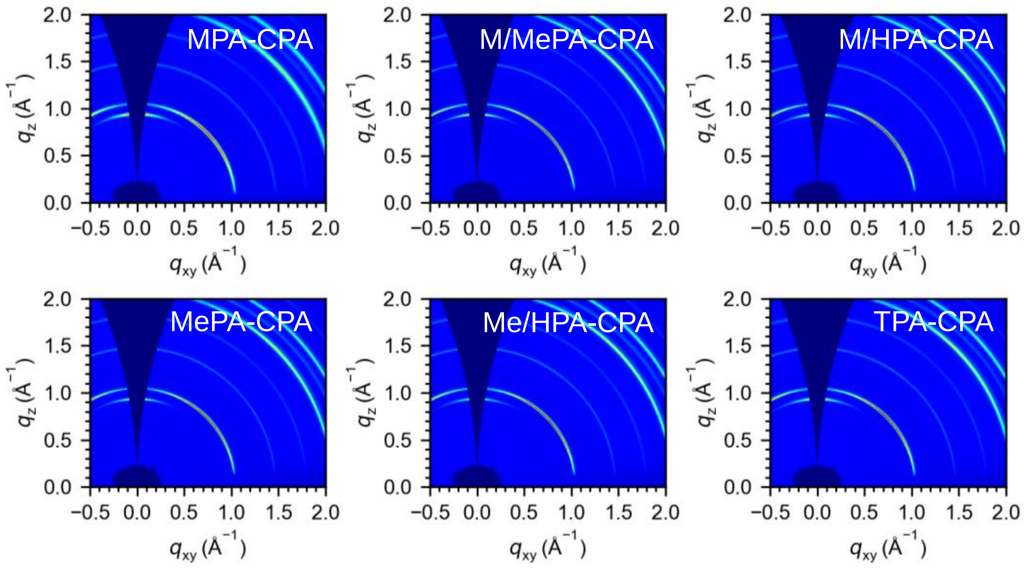

Figure S6. GIWAXS pattern of perovskite films deposited on different CPA-based HSMs.

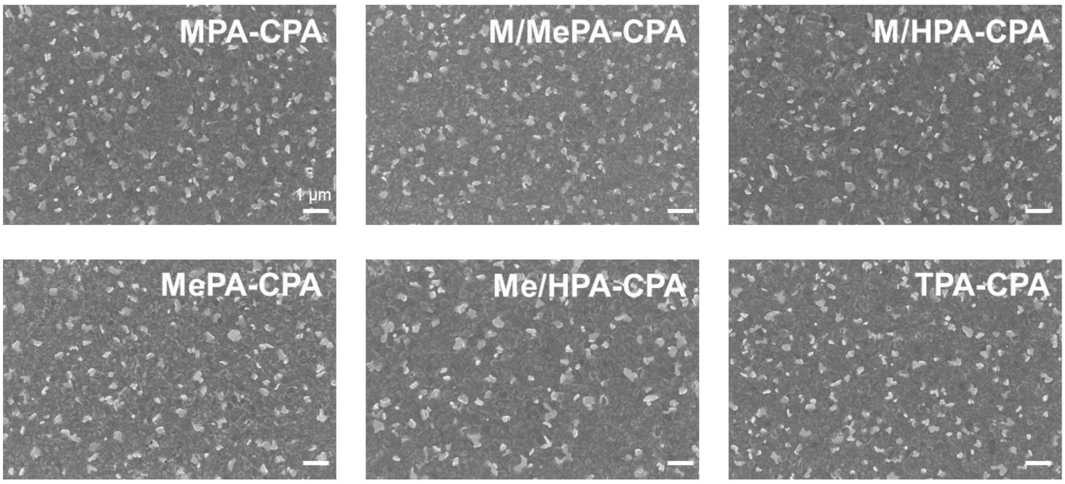

Figure S7. SEM images of the surface morphology of 1.76 eV perovskite films deposited on different HSMs.

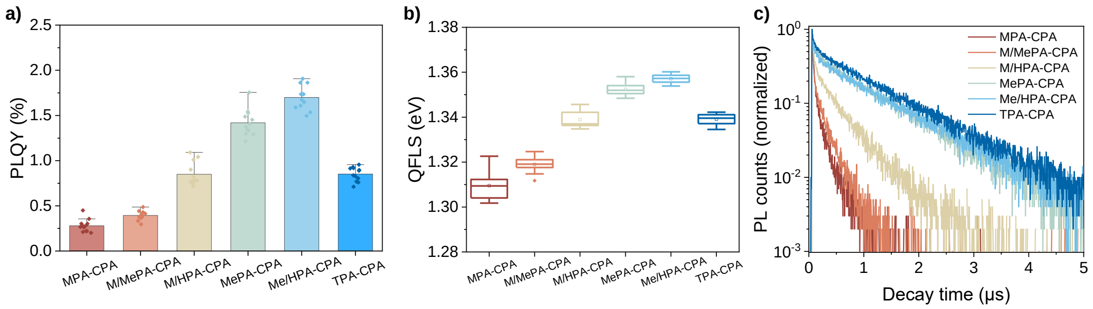

Figure S8. a) PLQY for the ITO/HSM/1.76 eV perovskite samples. b) QFLS of 1.76 eV perovskite films derived from PLQY values. The perovskite film deposited on **Me/HPA-CPA** achieved a notably highest QFLS, exceeding 1.36 eV, compared to **MPA-CPA** (1.31 eV), **M/MePA-CPA** (1.32 eV), **M/HPA-CPA** (1.34 eV), **MePA-CPA** (1.35 eV) and **TPA-CPA** (1.34 eV). c) Normalized photoluminescence decays of WBG perovskite films deposited on different HSMs.

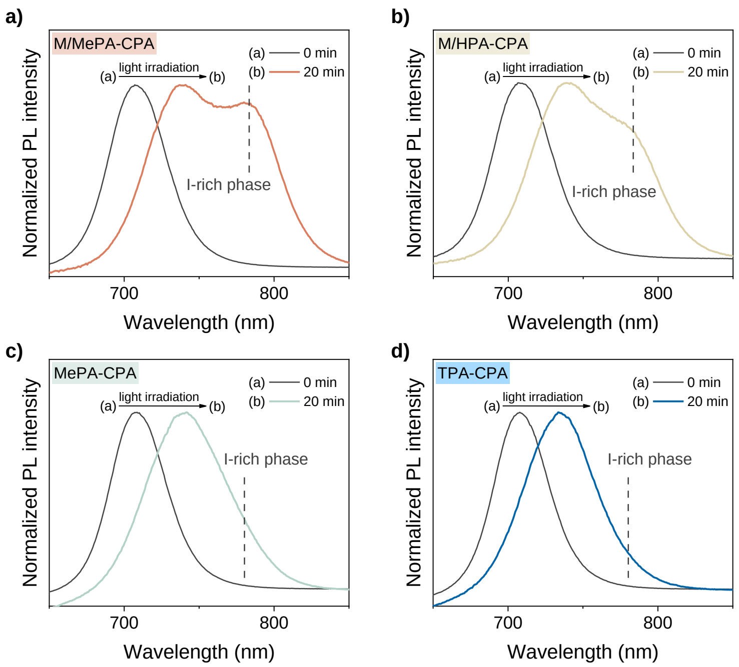

Figure S9. The PL spectra of 1.76 eV perovskite deposited on different HSMs before and after light irradiation for 20 minutes. We note that the double-peak emission appeared in perovskites deposited on **M/MePA-CPA** and **M/HPA-CPA**.

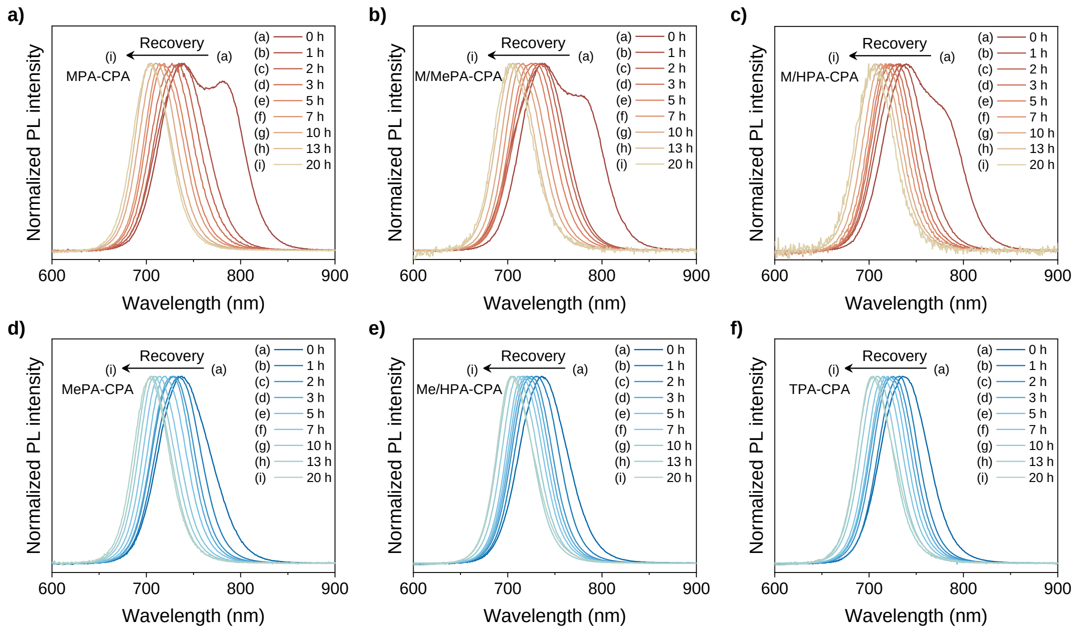

Figure S10. PL spectra of 1.76 eV perovskite deposited on CPA-based HSMs with different dark recovery times.

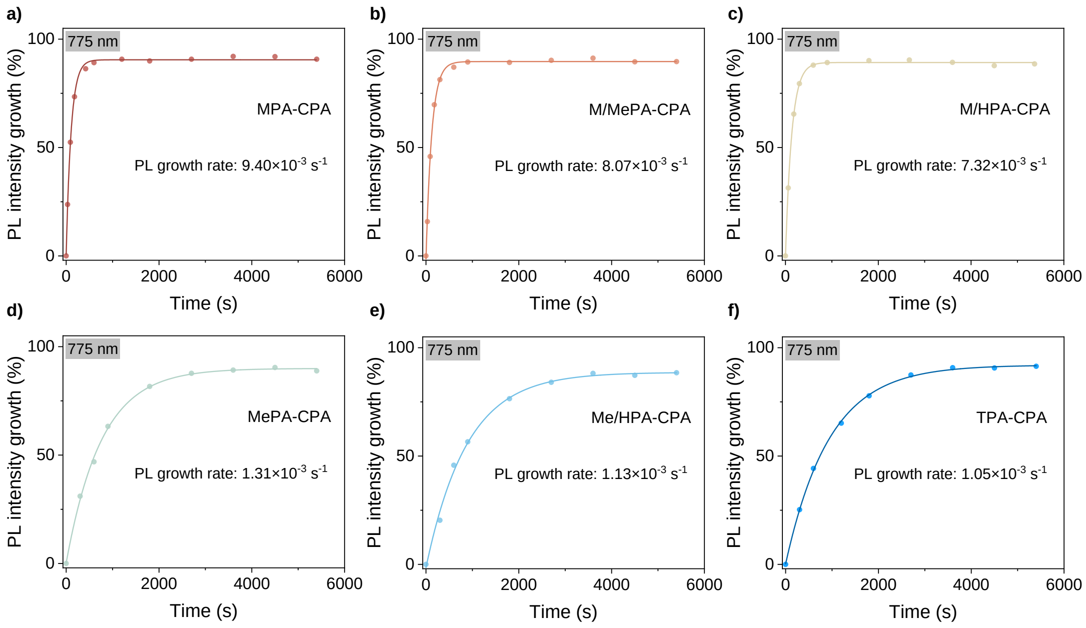

Figure S11. Trace of the PL intensity growth at the I-rich phase emission (775 nm) for perovskites deposited on CPA-based HSMs under 90 minutes light illumination (dots), and fitted using monoexponential kinetic (solid line).

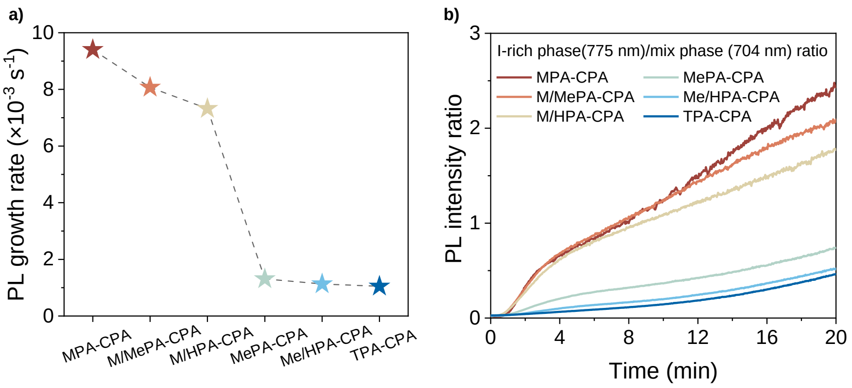

Figure S12. a) PL growth rate at 775 nm in perovskite films deposited on CPA-based HSMs. b) Time-dependent ratio of PL intensities at 775 nm (iodide-rich phase) and 704 nm (mixed perovskite phase) from the PL evolutions of perovskite films deposited on CPA-based HSMs.

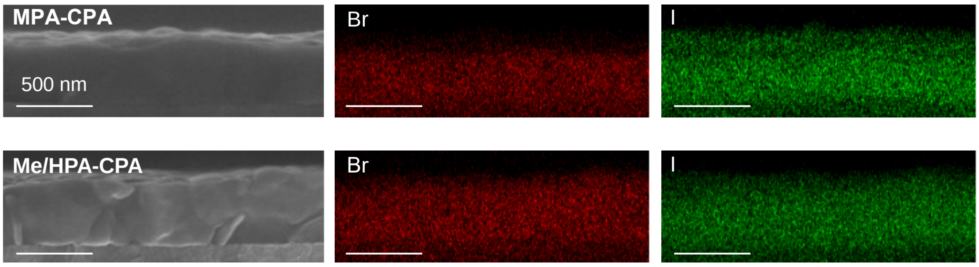

Figure S13. The cross-section SEM-EDS elemental mapping images of perovskite films deposited on **MPA-CPA** and **Me/HPA-CPA** after light irradiation for 30 minutes. Scale bar = 500 nm.

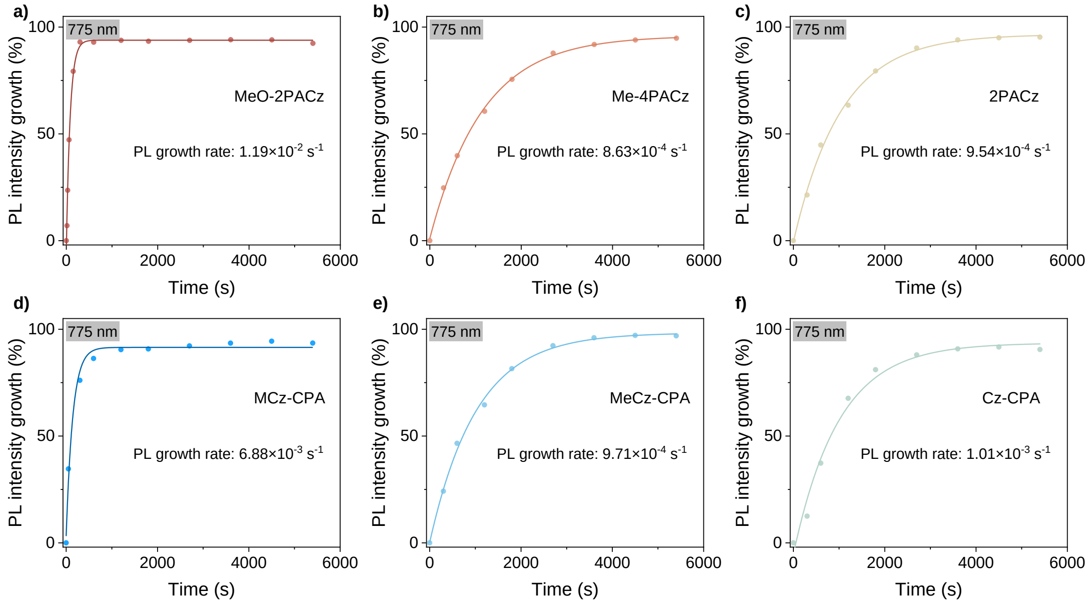

Figure S14. Trace of the PL intensity growth at 775 nm for perovskites deposited on carbazolyl-based (Cz-based) HSMs under 90 minutes light illumination (dots), and fitted using monoexponential kinetic (solid line).

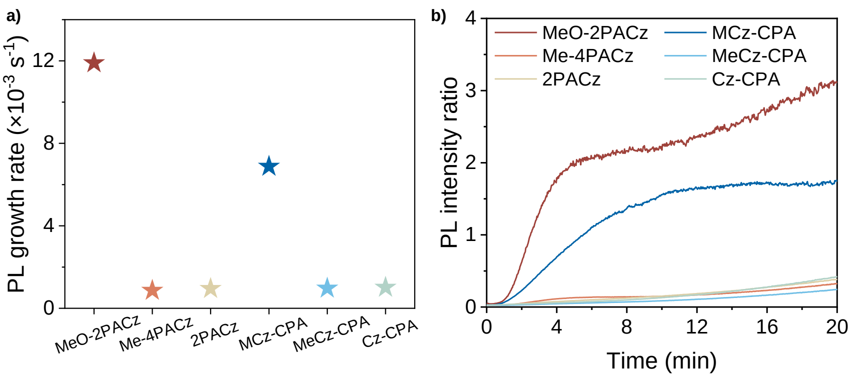

Figure S15. a) PL growth rate at 775 nm in perovskite films deposited on Cz-based HSMs. b) Time-dependent ratio of PL intensities at 775 nm (I-rich phase) and 704 nm (mixed perovskite phase) from the PL evolutions of perovskite films deposited on Cz-based HSMs.

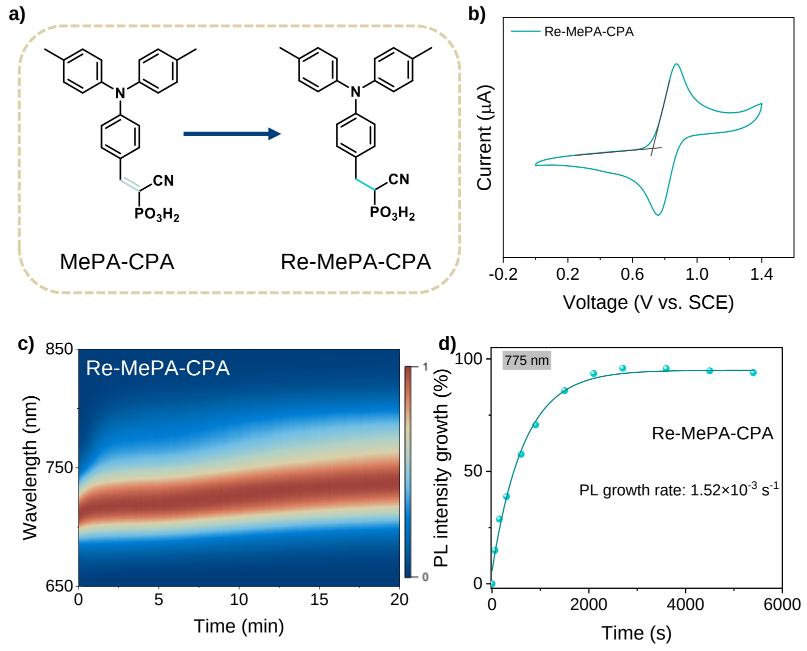

Figure S16. a) The molecular structure of **MePA-CPA** and **Re-MePA-CPA**. b) CV curves of **Re-MePA-CPA** measured in CH_2Cl_2, with Fc/Fc^+ serving as the external standard. The HOMO energy level of **Re-MePA-CPA** was calculated to be -5.47 eV. c) Evolution of PL spectra of 1.76 eV perovskite tracked over 20 minutes utilizing **Re-MePA-CPA** as HSM. The scale bar represents the normalized PL intensity of perovskite films. d) Trace of the PL intensity growth at 775 nm for perovskites under 90 minutes light illumination (dots), and fitted using monoexponential kinetic (solid line).

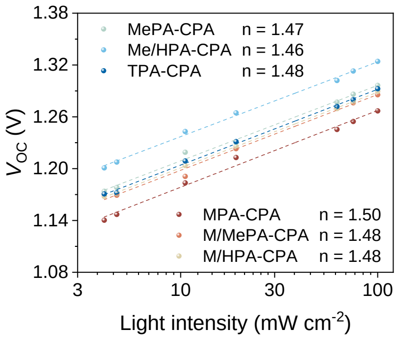

Figure S17. Light-intensity dependence of V_{OC} parameter of WBG perovskite devices based on different HSMs.

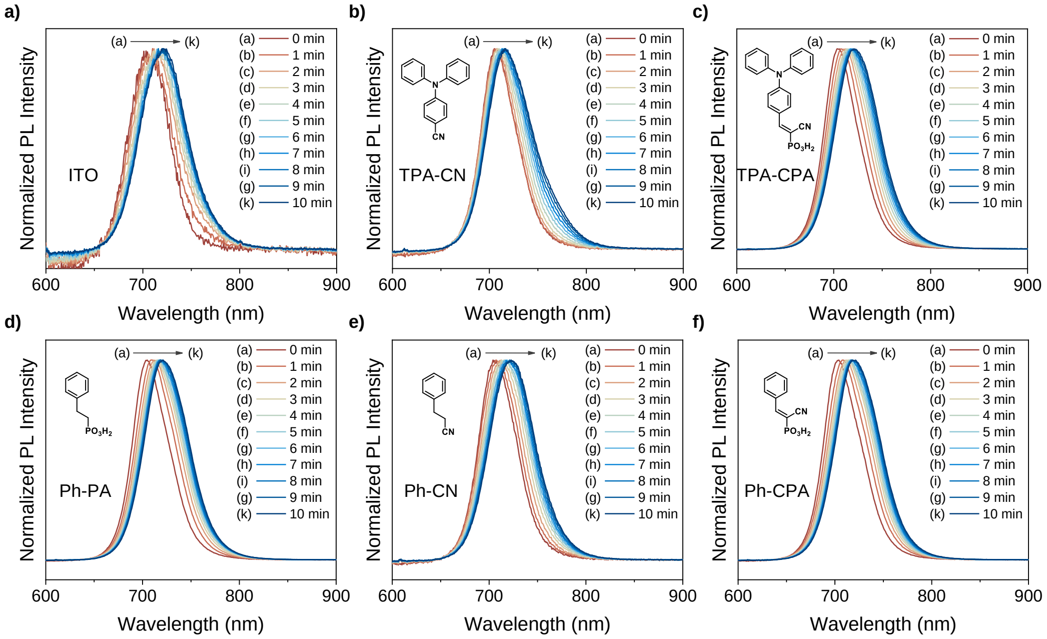

Figure S18. PL spectra evolution of perovskites deposited on different substrates under light irradiation for 10 minutes.

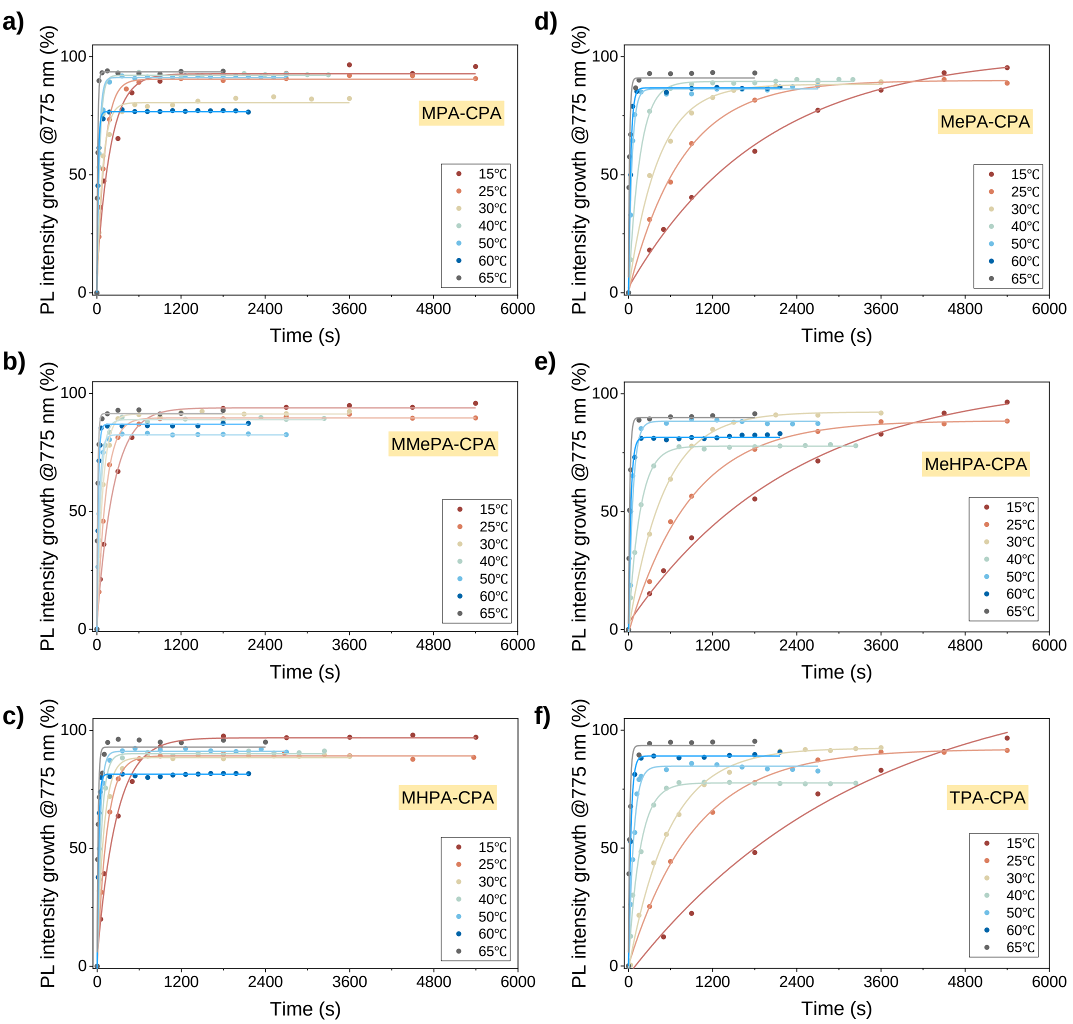

Figure S19. Trace of the PL intensity growth at 775 nm for perovskites deposited on CPA-based HSMs at different temperatures (dots), and fitted using monoexponential kinetic (solid line).

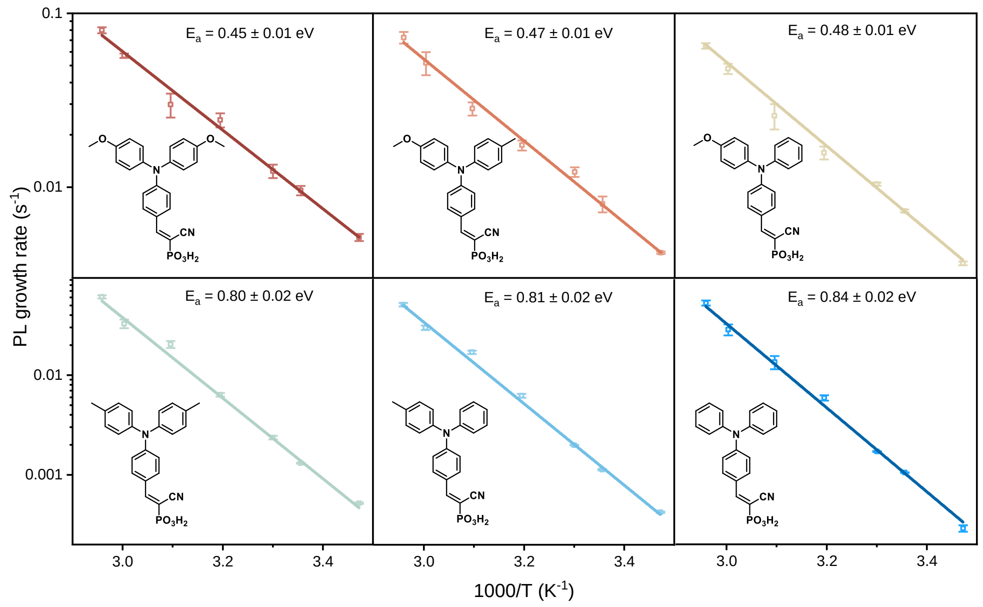

Figure S20. Temperature dependence of the PL growth rate. The PL growth rate (k) was extracted from the monoexponential fits in Figure S19. The activation energy (E_a) of photo-induced halide segregation (PIHS) in perovskite deposited on different HSMs were calculated through the Arrhenius equation: $\ln(k) = -\frac{E_a}{RT} + \ln(A)$. The E_a of PIHS in perovskites deposited on **MPA-CPA**, **M/MePA-CPA** and **M/HPA-CPA** are around 0.47 eV, and that on **MePA-CPA**, **Me/HPA-CPA** and **TPA-CPA** are around 0.81 eV.

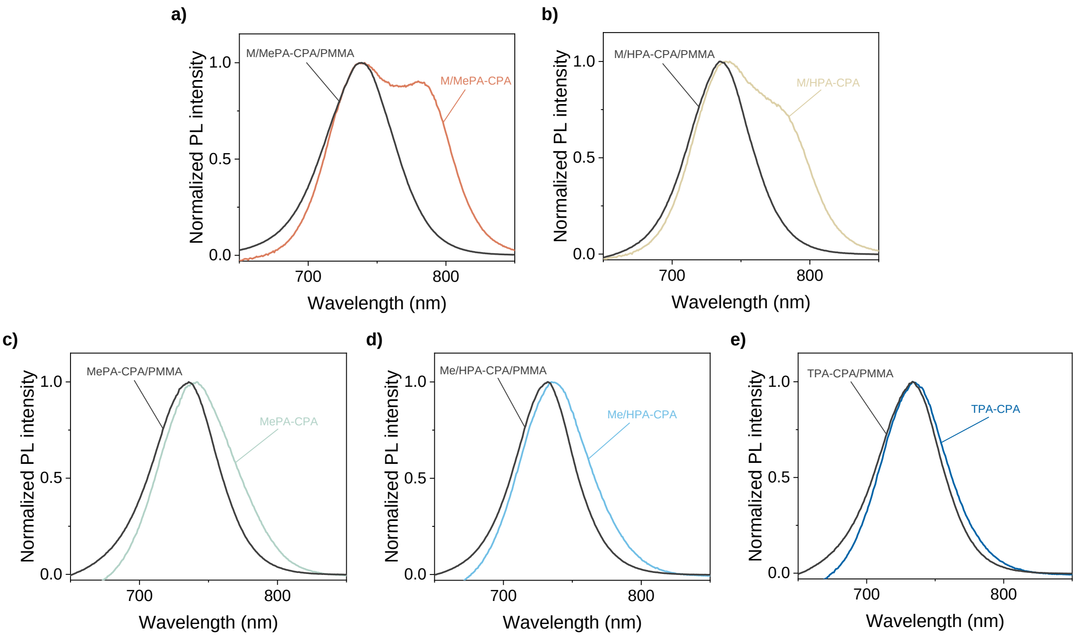

Figure S21. The PL spectra of 1.76 eV perovskite deposited on different HSMs with and without poly (methyl methacrylate) (PMMA) after light irradiation for 20 minutes. We note that applying PMMA as insert layer effectively suppress the PIHS of perovskite, especially for perovskite deposited on HSMs containing methoxy (MeO) substituents in molecules.

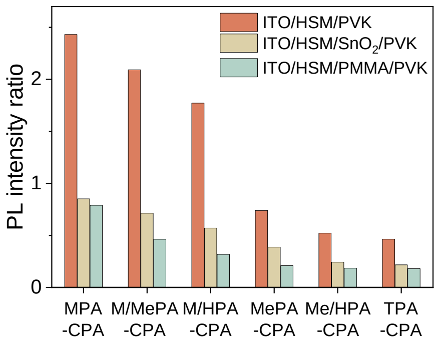

Figure S22. The PL intensity ratio of perovskites deposited on HSM, HSM/tin oxide (SnO_2), and HSM/PMMA after light irradiation for 20 minutes. We note that applying PMMA or SnO_2 deposited by atomic layer deposition technique as insert layer effectively suppress the PIHS of perovskite, especially for perovskite deposited on MeO group carrying HSMs.

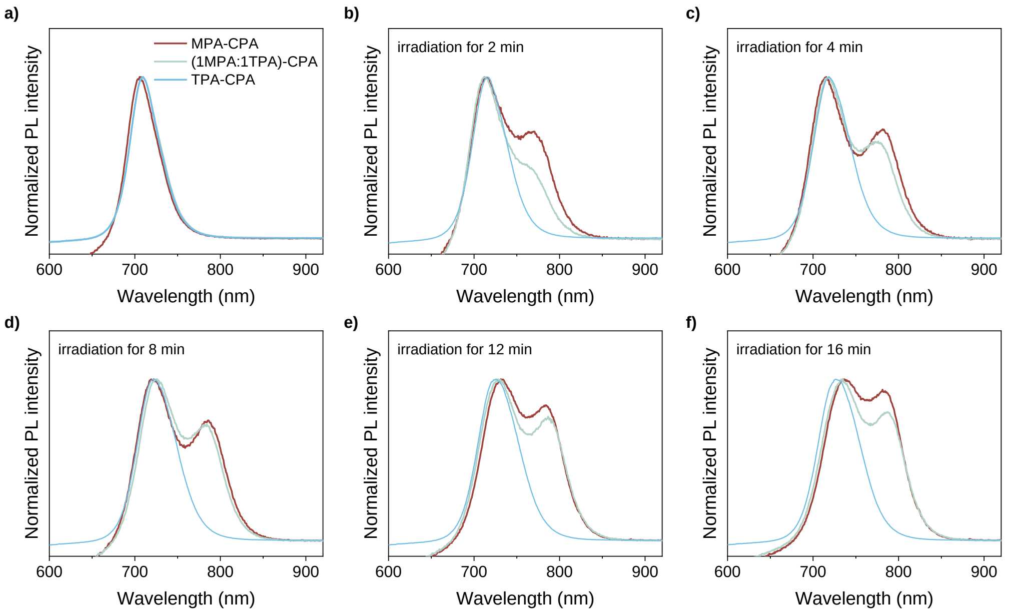

Figure S23. The PL spectra of 1.76 eV perovskite deposited on the mixed HSMs of **MPA-CPA** and **TPA-CPA** with various molar ratios under different light irradiation time. We note that the double-peak emission appeared in perovskites deposited on HSMs containing **MPA-CPA** (MeO substituent).

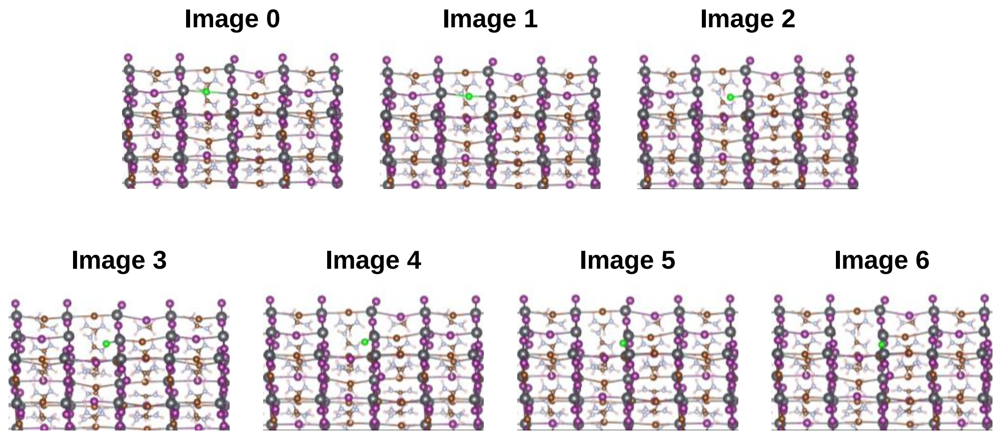

Figure S24. Images of simulations for migration of halide ion to adjacent halide vacancies in perovskite model.

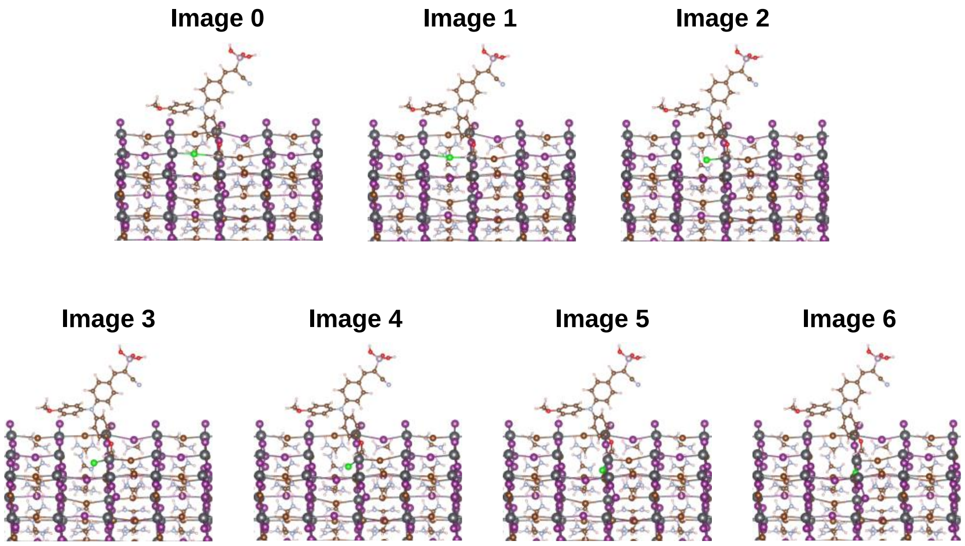

Figure S25. Images of simulations for migration of halide ion to adjacent halide vacancies in perovskite/**MPA-CPA** (containing MeO group) model, the MeO group of the molecule at a height of 1.554 Å above the top of the surface.

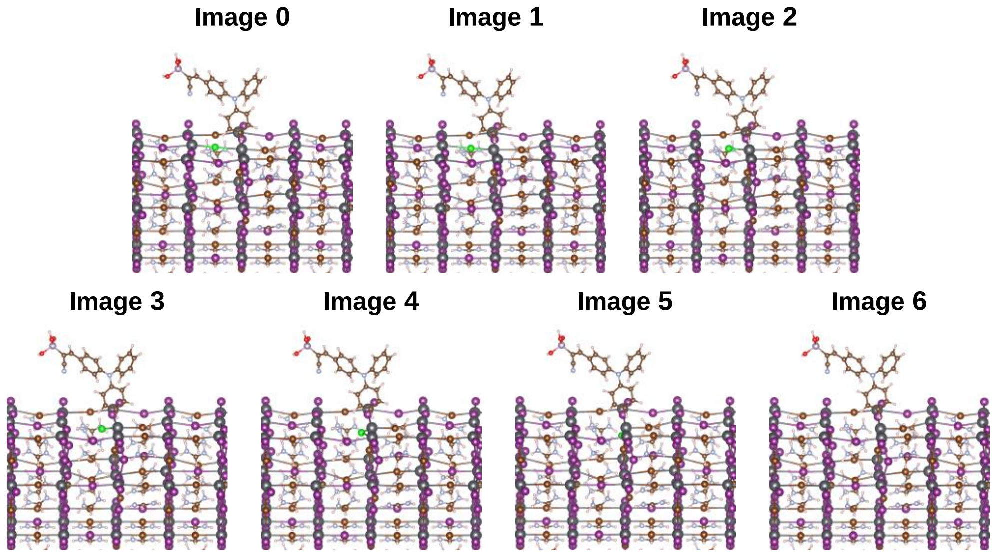

Figure S26. Images of simulations for migration of halide ion to adjacent halide vacancies in perovskite/**TPA-CPA** model.

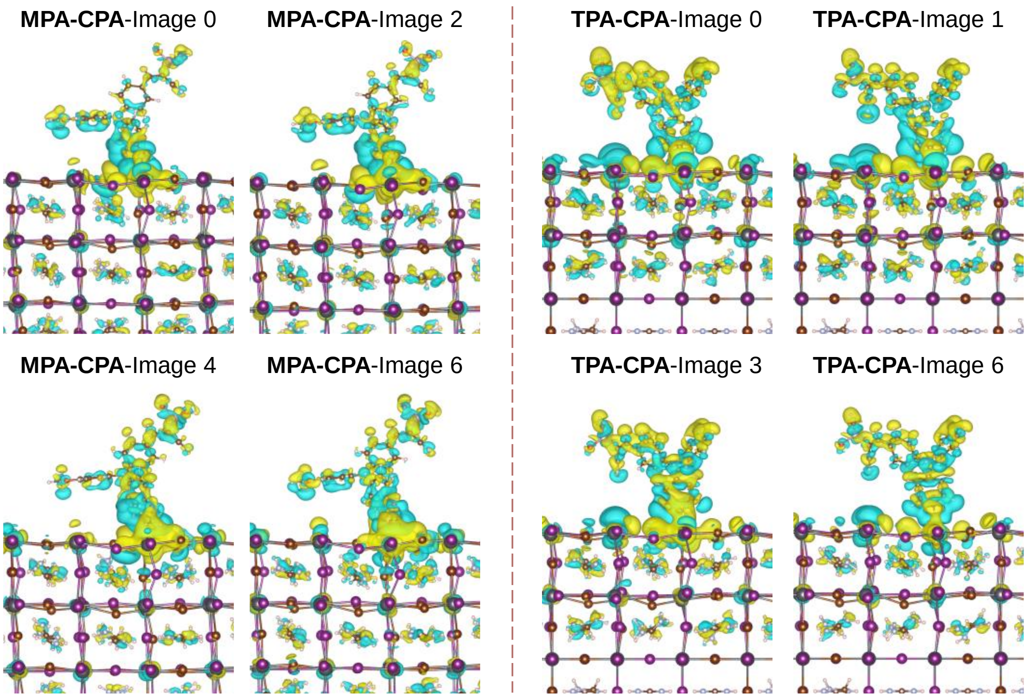

Figure S27. The plane-averaged charge density difference of **MPA-CPA** (left) and **TPA-CPA** (right) calculated by DFT in different images of simulations for halide ions migration.

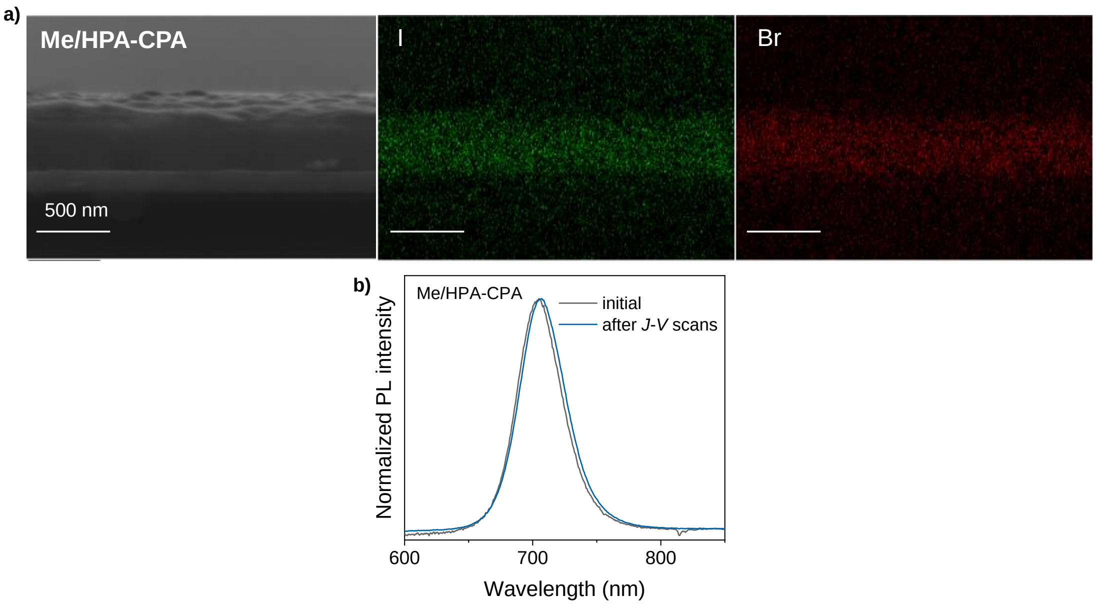

Figure S28. The a) cross-section elemental mapping images by SEM and b) PL spectra of **Me/HPA-CPA** based WBG-PSCs after repeated *J-V* scans. Scale bar = 500 nm.

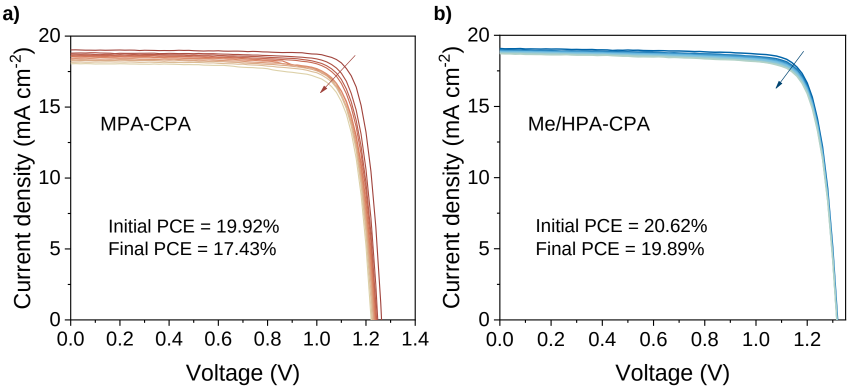

Figure S29. The *J–V* curves of 30 consecutive scanning cycles with a scan rate of 0.10 V s^{-1} for the WBG-PSCs based on a) **MPA-CPA** and b) **Me/HPA-CPA**.

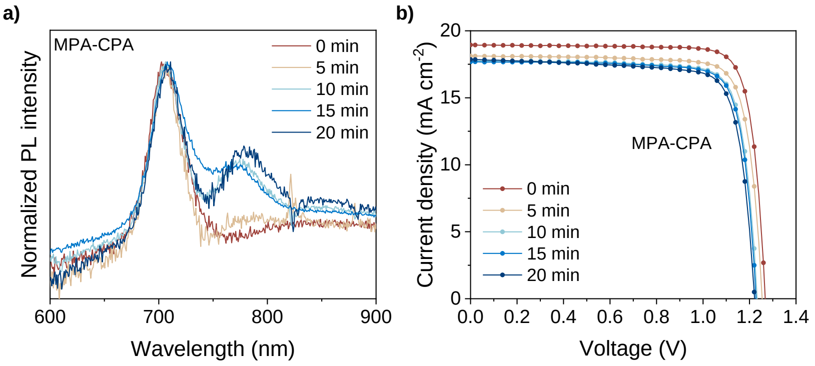

Figure S30. Evolution of a) PL spectra and b) *J–V* curves of **MPA-CPA** based devices tracked over 20 minutes under light irradiation (xenon lamp, 1 sun).

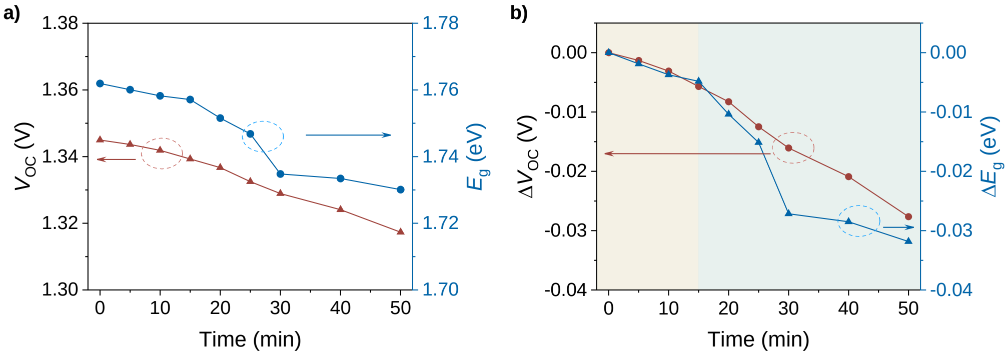

Figure S31. Evolution of V_{OC} and band-gap (E_g) of **Me/HPA-CPA** based devices tracked over 50 minutes under light irradiation.

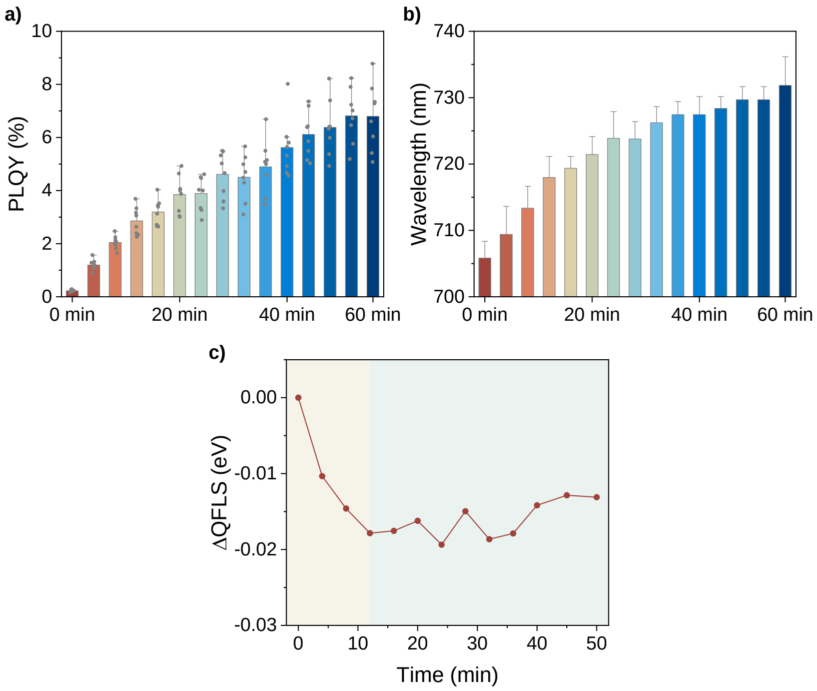

Figure S32. Evolution of PLQY, PL emission wavelength and ΔQFLS for ITO/**Me/HPA-CPA**/1.76 eV perovskite/passivation/C_{60} stacks under light irradiation.

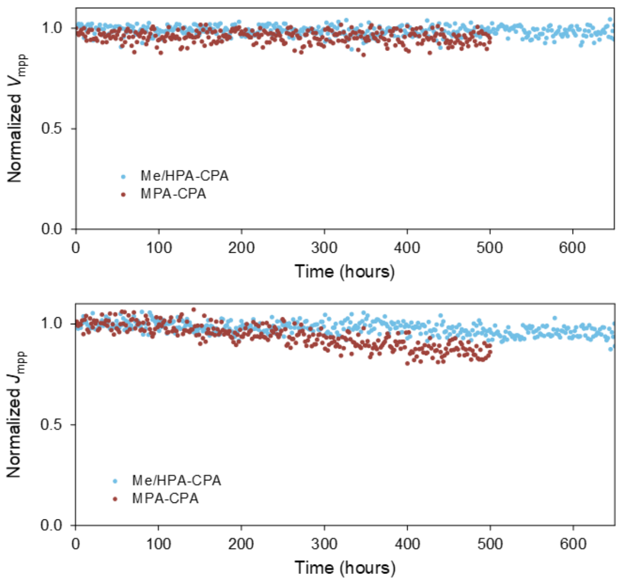

Figure S33. The normalized V_{mpp} and J_{mpp} (obtained from *J-V* characteristic) evolution of WBG devices based on **MPA-CPA** and **Me/HPA-CPA** during long-term MPP tracking. We evaluated the stability of WBG-PSCs in nitrogen with ISOS-L-1, under a white light-emitting diodes light with 1 sun equivalent intensity at ~45 °C.

Table S1. Summary of the optical and electrochemical properties of different HSMs.

	**λ_{max} [a] (nm)**	**E_g [b] (eV)**	**HOMO [c] (eV)**	**LUMO (eV)**
**MPA-CPA**	396	2.70	-5.46	-2.76
**M/MePA-CPA**	395	2.73	-5.53	-2.80
**M/HPA-CPA**	392	2.78	-5.54	-2.76
**MePA-CPA**	394	2.76	-5.59	-2.83
**Me/HPA-CPA**	391	2.81	-5.61	-2.80
**TPA-CPA**	388	2.83	-5.71	-2.88
**Re-MePA-CPA**	/	/	-5.47	/

[a] Measured in an ethanol solution at a concentration of 10^{-5} mol L^{-1}.

[b] Estimated from the solution's absorption edge using the equation: $E_g = 1240/\lambda_{onset}$ (eV).

[c] HOMO energy level was extracted from Figure S1 and Figure S16. The lowest unoccupied molecular orbital (LUMO) energy level was calculated using the E_g and HOMO values.

Table S2. Summary of photovoltaic parameters for WBG-PSCs employing different CPA-based HSMs, as presented in Figure S3.

	**V_{OC} (V)**	**J_{SC} (mA cm^{-2})**	**FF (%)**	**PCE (%)**
**MPA-CPA**	1.262	19.16	80.06	19.36
**M/MePA-CPA**	1.284	19.12	81.70	20.07
**M/HPA-CPA**	1.292	19.23	81.99	20.38
**MePA-CPA**	1.299	19.14	82.96	20.64
**Me/HPA-CPA**	1.319	19.02	83.71	20.99
**TPA-CPA**	1.293	19.02	82.79	20.36

Table S3. Compilation of PLQY and QFLS data for ITO/HSMs/perovskite samples, along with the τ_{SRH} values for perovskite films utilizing various HSMs.

	**PLQY (%)**	**QFLS (eV)**	**τ_{SRH}(μs)**
**MPA-CPA**	0.279±0.070	1.310±0.006	0.23
**M/MePA-CPA**	0.394±0.050	1.319±0.003	0.24
**M/HPA-CPA**	0.849±0.129	1.339±0.004	0.49
**MePA-CPA**	1.419±0.146	1.352±0.003	0.94
**Me/HPA-CPA**	1.699±0.132	1.357±0.002	1.09
**TPA-CPA**	0.851±0.078	1.339±0.002	1.10

Table S4. Summary of the PL growth rate (k) for different perovskite films calculated from Figure S19 and fitted using monoexponential kinetic.

**k ($\times 10^{-3}$ s^{-1})**	**15°C**	**25°C**	**30°C**	**40°C**	**50°C**	**60°C**	**65°C**
**MPA-CPA**	5.14	9.57	12.38	24.33	29.85	57.08	80.04
**M/MePA-CPA**	4.20	8.00	12.23	17.43	28.29	51.96	72.57
**M/HPA-CPA**	3.64	7.30	10.41	15.78	25.76	47.97	65.02
**MePA-CPA**	0.52	1.32	2.37	6.36	20.38	32.88	61.12
**Me/HPA-CPA**	0.42	1.14	2.00	6.26	17.08	30.10	51.28
**TPA-CPA**	0.29	1.05	1.71	5.90	13.47	28.54	52.68

References

1. G. Kresse and J. Furthmüller, *Computational Mater. Sci.*, 1996, **6**, 15-50.
2. G. Kresse and J. Furthmüller, *Physical Review*, 1996, **54**, 11169-11186.
3. J. P. Perdew, K. Burke and M. Ernzerhof, *Erratum Phys. Rev. Lett.*, 1996, **77**, 3865-3868.
4. G. Henkelman, B. P. Uberuaga and H. Jónsson, *J. Chem. Phys.*, 2000, **113**, 9901–9904.
